# Ginsenoside Rg1 as a Potential Regulator of Hematopoietic Stem/Progenitor Cells

**DOI:** 10.1155/2021/4633270

**Published:** 2021-12-31

**Authors:** Fang He, Guanping Yao

**Affiliations:** ^1^Key Laboratory of Cell Engineering in Guizhou Province, The Affiliated Hospital of Zunyi Medical University, Zunyi, Guizhou, China; ^2^Department of Reproductive Medicine Center, The Affiliated Hospital of Zunyi Medical University, Zunyi, Guizhou, China

## Abstract

Ginsenoside Rg1 (Rg1), a purified, active component of the root or stem of ginseng, exerts positive effects on mesenchymal stem cells (MSCs). Many recent studies have found that hematopoietic stem cells (HSCs), which can develop into hematopoietic progenitor cells (HPCs) and mature blood cells, are another class of heterogeneous adult stem cells that can be regulated by Rg1. Rg1 can affect HSC proliferation and migration, regulate HSC/HPC differentiation, and alleviate HSC aging, and these findings potentially provide new strategies to improve the HSC homing rate in HSC transplantation and for the treatment of graft-versus-host disease (GVHD) or other HSC/HPC dysplasia-induced diseases. In this review, we used bioinformatics methods, molecular docking verification, and a literature review to systematically explore the possible molecular pharmacological activities of Rg1 through which it regulates HSCs/HPCs.

## 1. Introduction

**Figure 1:**
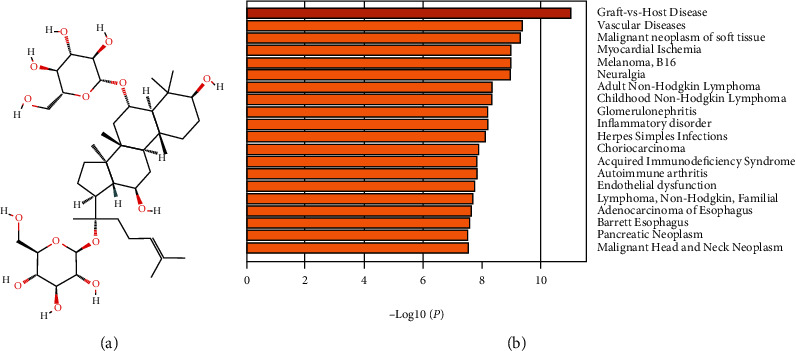
Molecular structure of Rg1 and the diseases that may be targeted by Rg1. (a) Molecular structure of ginsenoside Rg1. (b) GVHD is the top putative disease targeted by Rg1, as shown by an analysis using the Metascape platform.

Ginsenosides are the active components of ginseng and comprise a group of sterol compounds. According to differences in their glycosidic structure, ginsenosides are divided into two subtypes: the dammarane type and the oleanane type [1, 2]. Ginsenoside Rg1 (Rg1, molecular formula: C_42_H_72_O_14_, Figure 1(a), image from PubChem), a member of the ginsentriol subtype of dammarane ginsenosides, is an important monomeric ginsenoside and the most abundant component of Chinese/Korean ginseng. Rg1 not only acts on the nervous, cardiovascular, blood, and immune systems but also exhibits a variety of positive pharmacological activities, such as its neuroprotective activity [3] and its abilities to treat myocardial ischemia [4] and repair hematopoietic immune disorders [5]. To date, several clinical trials on the use of drugs containing Rg1 for the treatment of vascular dementia, hyperlipidemia, hypertension, Sjögren's syndrome, rheumatic diseases, and ischemic stroke have been registered on clinicaltrials.gov [6, 7].

Hematopoietic stem cells (HSCs) are adult stem cells that can self-renew, differentiate into blood cell lineages, and exert long-term effects on maintaining and producing all mature blood cell lineages during the life cycle of an organism [8]. Under a stable metabolism status, most HSCs are in a static state (quiescent HSCs), whereas hematopoietic progenitor cells (HPCs) actively proliferate and maintain the daily hematopoietic function. When the body is stimulated, such as during life-threatening blood loss, infection, and inflammation, HSCs can be activated in the bone marrow to proliferate and participate in blood formation [9, 10]. HSCs mainly function in the specific bone marrow microenvironment (HSC niche), which provides the signals needed to protect HSCs and maintain HSC differentiation [11]. Although the surface phenotypes of human and murine HSCs differ, these cell types possess the basic functions of HSCs. CD34 is a marker of human HSCs, and clinical transplantation studies using enriched CD34^+^ bone marrow cells have indicated the presence of HSCs with the ability to reconstitute bone marrow within this fraction [12, 13]. For differentiation and functional research involving murine HSCs, a variety of markers are commonly used to identify or isolate HSCs. In general, murine HSCs do not express lineage-specific markers (Lin^–^) and are positive for c-kit (CD117^+^ or c-Kit^+^) and stem cell antigen-1 (Sca-1^+^). More recently, the HSC population was further clarified to include the markers CD150 and CD48, and as a result, this population was defined by the marker profile Lin^–^c-Kit^+^Sca-1^+^CD48^–^CD150^+^ [14–16].

In a previous study, we described the active regulatory effects of Rg1 on the proliferation, differentiation, senescence, and apoptosis of mesenchymal stem cells (MSCs) [17]. Here, HSCs, a heterogeneous adult stem cell population that completely differs from MSCs, were analyzed and systematically reviewed. We used bioinformatics methods to analyze the potential molecular pharmacological role of Rg1 in HSC/HPC regulation and reviewed the literature to summarize the mechanisms through which Rg1 activates HSC proliferation and differentiation and its antiaging effects in HSCs/HPCs.

## 2. Prediction of Potential Rg1 Targets in HSCs/HPCs Based on a Bioinformatics Analysis

The molecular structure of Rg1 from PubChem was analyzed to identify putative targets of Rg1, and the TargetNet and SwissTargetPrediction platforms were also used to predict putative targets of the ginsenoside Rg1 [18, 19]. A comprehensive search identified 723 putative targets (623 from TargetNet and 100 from SwissTargetPrediction) of Rg1 (Supplementary Materials 1 and 2). Furthermore, the top 20 targets (10 from TargetNet and 10 from SwissTargetPrediction) with relatively high probability among the putative targets were pooled and used to predict associated diseases that may be regulated by Rg1 (analyzed by DisGeNET using the Metascape platform [20]). The results showed that graft-versus-host disease (GVHD) is the top putative targeted disease that may be regulated by Rg1 (Figure 1(b)). This finding suggests that Rg1 could be used as a potential monomeric drug to reduce GVHD and that Rg1 could further improve the success rate of HSC transplantation.

**Figure 2:**
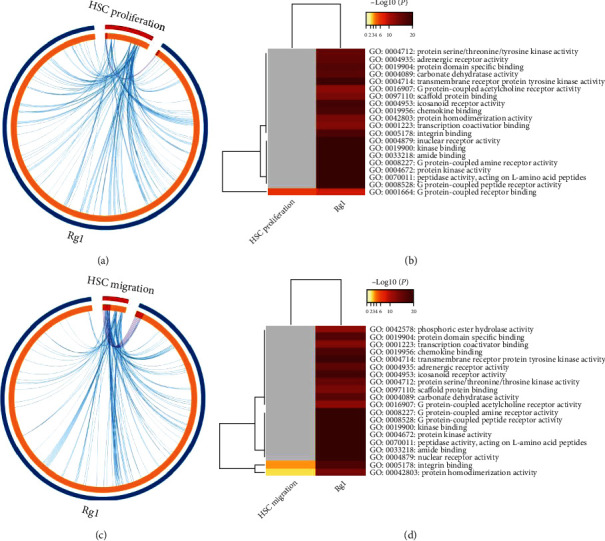
Metaenrichment analysis of the molecular effects of Rg1 on HSC proliferation or migration. (a) Ring summary shows overlapping genes related to Rg1 and HSC proliferation at the gene level (the purple line shows the overlapping genes; the blue line shows a functional correlation between genes). (b) Heatmap of terms enriched in the list of genes targeted by Rg1 to regulate HSCs. The terms are colored based on the *p* value. (c) Ring summary of overlapping genes through which Rg1 regulates HSC migration at the genetic level. (d) Heatmap of terms enriched in the genes targeted by Rg1 to regulate HSC migration. The terms are colored based on the *p* value.

To perform an interactive bioinformatics analysis of the relationships between HSC proliferation and migration and Rg1, we analyzed the molecular functions of Rg1 through a metaenrichment of pathway. Genes that may be related to HSC proliferation or migration were analyzed using the Comparative Toxicogenomics Database, and we identified 49 genes associated with HSC proliferation and 23 genes (after removing repetition) associated with HSC migration (Supplementary Materials 3 and 4). A metaenrichment analysis of Rg1 and HSC targets was performed using the Metascape platform, and the results showed that G protein-coupled receptor binding was enriched in the effects of Rg1 on HSC proliferation, whereas integrin binding and protein homodimerization activity were enriched in the effects of Rg1 on HSC migration (Figure 2).

**Figure 3:**
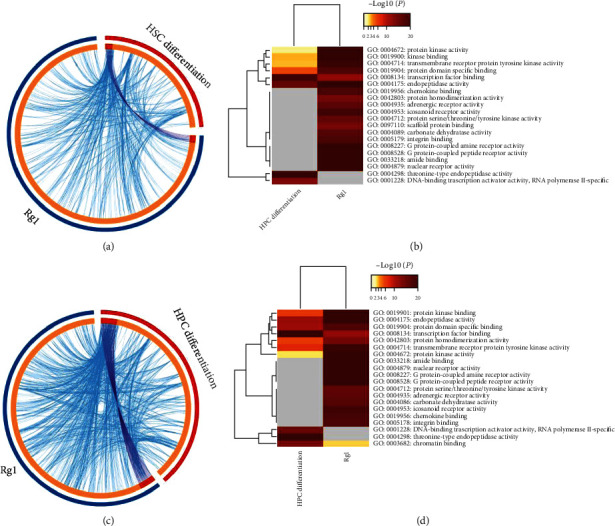
Metaenrichment analysis of the molecular effects of Rg1 on HSC/HPC differentiation. (a) Ring summary of overlapping genes through which Rg1 regulates HSC differentiation at the genetic level (the purple line shows overlapping genes; the blue line shows a functional correlation between genes). (b) Heatmap of terms enriched in the genes targeted by Rg1 to regulate differentiation. The terms are colored based on the *p* value. (c) Ring summary of overlapping genes through which Rg1 regulates HPC differentiation at the genetic level. (d) Heatmap of terms enriched in the genes targeted by Rg1 to regulate HPCs differentiation. The terms are colored based on the *p* value.

In addition, we used bioinformatics methods to analyze the potential molecular biological functions of Rg1 during the process of HSC or HPC differentiation. Putative targets of HSC or HPC differentiation were obtained using the Comparative Toxicogenomics Database. A total of 183 and 359 genes (after removing repetition) related to HSC and HPC differentiation, respectively, were identified (Supplementary Materials 5 and 6). The results of the metaenrichment analysis of pathways using the Metascape platform showed that transcription factor binding and endopeptidase activity are enriched molecular functions in HSC differentiation that may be regulated by Rg1. Moreover, protein domain-specific binding was found to be an additional molecular function that may be regulated by Rg1 during HPC differentiation (Figure 3).

**Figure 4:**
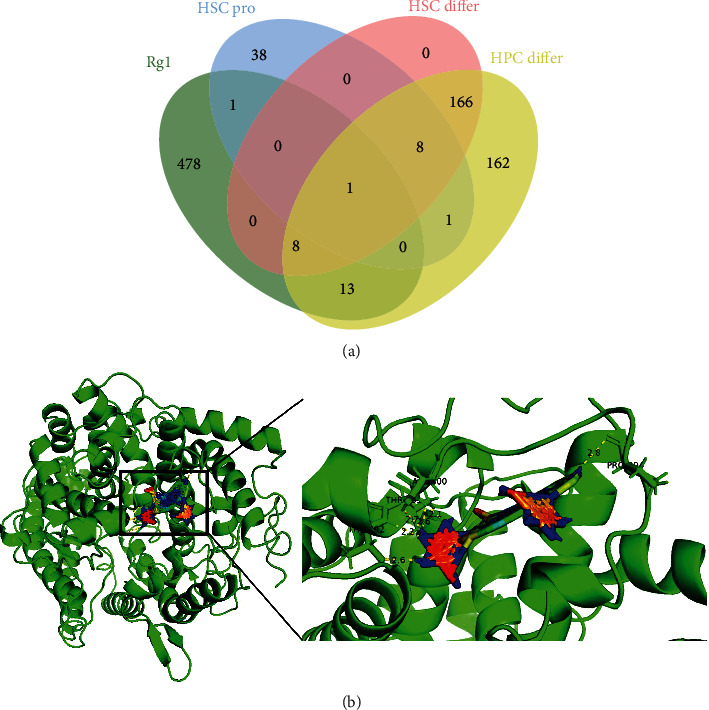
Common proteins among putative proteins targeted by Rg1 and proteins related to HSC proliferation, HSC differentiation, and HPC differentiation. (a) Venn diagram showing that one protein (ACE) was found in all four lists. (b) Three-dimensional schematic representation showing the molecular docking model, active sites, and binding distances for Rg1 and ACE after the application of ray tracing.

Interestingly, the use of JVenn to visualize the specific targets through which Rg1 regulates HSCs [21] revealed that angiotensin-converting enzyme (ACE, GeneID: 1636) was the only overlapping gene through which Rg1 regulates HSC proliferation and HSC/HPC differentiation (Figure 4). We then used molecular docking to verify the interaction between Rg1 and ACE. Briefly, the crystal structures of putative targets were obtained from the Protein Data Bank, and AutoDock Tools 1.5.6-Vina software was used for the analysis of binding ability and sites. Additionally, PyMOL was utilized to visualize the interaction between Rg1 and the ACE peptide chain. The results showed that several hydrogen bonds may form between Rg1 and ACE. Specifically, the use of the minimum binding energy (affinity: -13.0 kcal/mol) in the docking analysis revealed that hydrogen bond formed between Rg1 and threonine in chain A of ACE (Figure 4).

Furthermore, the signal pathways and molecular mechanism in the bioinformatics results provide some novel research directions and may be worth further exploration in in vitro/in vivo experiments. All the databases used in the study are listed in Supplementary Material 7.

## 3. A Review of the Literature Reveals That Rg1 Regulates HSC Proliferation, Differentiation, and Migration

**Figure 5:**
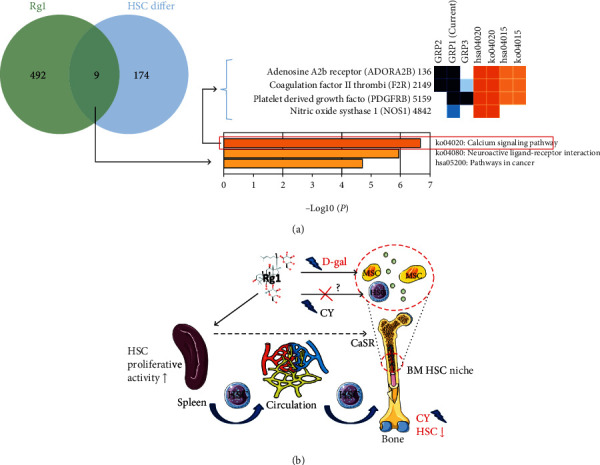
Relevant bioinformatics data and mechanistic summary of the mechanism through which Rg1 regulates the proliferation, differentiation, and migration of HSCs. (a) Venn diagram and KEGG pathway enrichment analyses revealed that the calcium signaling pathway is the key pathway through which Rg1 regulates HSC differentiation. (b) Rg1 promotes the homing of HSCs from the spleen to the bone marrow through the peripheral circulatory system in mice with myelosuppression caused by cyclophosphamide. CY: cyclophosphamide; HSC: hematopoietic stem cell; D-gal: D-galactose; CaSR: calcium-sensing receptor; MSC: mesenchymal stem cell.

HSCs differentiate into myeloid progenitor cells and prolymphoid progenitor cells in the bone marrow to drive bone marrow hematopoiesis [11, 22]. A previous study found that Rg1 can regulate calcium-sensing receptor (CaSR) to increase the number of Lin^–^Sca-1^+^c-Kit^+^ HSCs and lymphoid CD3^+^ cells in the bone marrow and peripheral blood of CY-induced myelosuppressed mice and thereby restores bone marrow function [23]. Interestingly, an analysis of the 9 overlapping target genes through which Rg1 regulates HSC differentiation using the Metascape platform revealed that the calcium signaling pathway was the key KEGG pathway through which Rg1 regulates HSC differentiation (Figure 5(a)). This consistent finding confirms that the CaSR-mediated calcium signaling pathway may be a crucial target through which Rg1 regulates HSC differentiation.

In addition, some studies have shown that Rg1 improves the hematopoietic activity of the bone marrow through extramedullary hematopoiesis. Cyclophosphamide (CY) can cause bone marrow cytotoxicity, leading to bone marrow suppression and triggering extramedullary hematopoiesis [24, 25]. Extramedullary hematopoiesis is characterized by the presence of pluripotent HPCs, including erythroid lineage cells, myeloid lineage cells, and megakaryocytes, in the spleen and liver [26]. Liu et al. [27] found that Rg1 treatment could effectively reduce the weight of the spleen of CY-stimulated mice and reduce the absolute number of c-Kit^+^ HSCs in the spleen and that these effects are not caused by apoptosis, which suggests that Rg1 alleviates CY-induced extramedullary hematopoiesis in the spleen. Further research shows that Rg1 could upregulate the proliferative activity of c-Kit^+^ HSCs in the spleen but not in the bone marrow of CY-stimulated mice. Moreover, Rg1 increases the number of c-Kit^+^/CD45^+^ HSCs in the peripheral circulatory system. Most importantly, the effect of Rg1 on HSCs in the bone marrow and peripheral blood is not observed in splenectomy- and CY-induced mice. These results systematically indicate that Rg1 improves CY-induced myelosuppression by activating HSC proliferation in the spleen, particularly by allowing the homing of HSCs from the spleen through the circulatory system to the bone marrow [27]. In addition, the selective regulation of HSCs in the spleen but not in the bone marrow also suggested that the “spleen-bone marrow” axis homing of HSCs plays a main/crucial role in Rg1 relieving extramedullary hematopoiesis and myelosuppression. Moreover, quiescent HSCs in bone marrow HSC “niche” can maintain hematopoietic homeostasis [28]. In the above study, after the new “niche” formed, the homing HSCs derived from the spleen may also serve as quiescent HSCs and further benefit bone marrow hematopoietic homeostasis.

Interestingly, whether Rg1 directly activates and promotes the proliferation of quiescent HSCs in the bone marrow niche was an open question. First, CD34^+^ cells account for only 1.5% of human bone marrow mononuclear cells, and murine Lin^−^Sca-1^+^c-Kit^+^ HSCs account for less than 1% of bone marrow cells. The treatment of mice with CY results in the appearance of a large amount of vacuole-like degradation in the bone marrow cavity and a sharp decrease in the number of bone marrow cells (including MSCs) [23, 29]. In this context, very few HSCs remain in the bone marrow, and the effect of Rg1 on enhancing the mobilization of extramedullary hematopoiesis far exceeds the effect of Rg1 on the mobilization of HSCs in the bone marrow. Therefore, the direct effect of Rg1 on quiescent HSCs in the bone marrow may be difficult to observe. Second, the researchers continuously administered Rg1 (15 mg/kg/day) to CY-induced myelosuppressed mice (splenectomy) for 7 days [27], and the results showed that Rg1 could not effectively increase the percentage of bone marrow Lin^−^Sca-1^+^c-Kit^+^ HSCs (no significant difference), but an increasing trend was observed. Third, Rg1 could also regulate MSCs to protect HSCs from D-galactose- (D-gal-) induced damage [30], and the continuous administration of Rg1 for 7 days could not completely restore the histological morphology of the murine bone marrow (vacuolar pathological structures remained in the bone marrow cavity) [23]. Thus, we infer that the regulatory effect of MSCs on the hematopoietic microenvironment could be delayed. Therefore, further investigation of whether Rg1 can activate HSCs in the bone marrow by prolonging the duration of Rg1 administration to explore the recovery of bone marrow HSCs in mice with splenectomy is warranted (Figure 5(b)).

In addition, stromal-derived factor-1 (SDF-1)/C-X-C chemokine receptor type 4 (CXCR4) is an important signaling molecule in HSC homing to the bone marrow and bone marrow implantation [31]. Rg1 can regulate the SDF-1*α*/CXCR4 axis and plays a regulatory role in the vascular intima [32]. These findings also suggest that Rg1 promotes HSC homing from the spleen to the bone marrow cavity and exerts hematopoietic effects in the bone marrow.

## 4. Mechanisms Involved in the Attenuation of HSC Aging by Rg1

**Figure 6:**
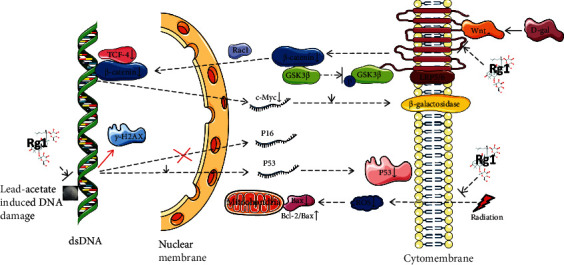
Rg1 alleviates HSC aging by regulating the Wnt/*β*-catenin and p53 signaling pathways. D-gal: D-galactose; LRP: low-density lipoprotein receptor-related protein; GSK-3*β*: glycogen synthase kinase 3 beta; Rac1: Ras-related C3 botulinum toxin substrate 1; TCF-1: transcription factor 4.

Traditionally, aging HSCs gradually lose the potential for self-renewal and differentiation, and the likelihood of abnormal metabolic cellular functions greatly increases [33]. An increasing number of studies have shown that inflammation and chemical or physical factors also cause DNA damage, which can lead to HSC aging [10, 34, 35]. Excessive D-gal results in the production of aldohexose and hydrogen peroxide via galactose oxidase and promotes the generation of oxygen-derived free radicals and superoxide anions, which results in impairment of the functions of macromolecules and cells [36, 37]. Rg1 inhibits oxidative stress and reduces DNA damage, which results in enhancement of the antiaging ability of Sca-1^+^HSCs/HPCs in a murine model of D-gal-induced aging, and the effect is related to inhibition of excessive activation of the Wnt/*β*-catenin signaling pathway. The classic Wnt/*β*-catenin pathway is essential for the regulation of stem cell pluripotency and the determination of cell fate [38, 39]. When D-gal activates the Wnt/*β*-catenin pathway, the Wnt ligand (a secreted glycoprotein that binds to Frizzled receptors) forms a large cell surface complex with low-density lipoprotein receptor-related protein (LRP) 5/6. A previous study found that Rg1 can inhibit D-gal-induced overactivation of the Wnt/*β*-catenin signaling pathway. Rg1 can reduce *β*-catenin expression and glycogen synthase kinase 3 beta (GSK3*β*) phosphorylation in the cytoplasm and can further reduce the protein expression of *β*-catenin in the nucleus via Ras-related C3 botulinum toxin substrate 1 (Rac1) and other factors; in addition, the binding of *β*-catenin to the transcription factor TCF-4 in the nucleus is reduced and ultimately inhibits c-Myc gene expression, which results in the reduction of *β*-galactosidase expression [40] (Figure 6).

A previous study also showed that Rg1 could mediate the p53-p21-Rb signaling pathway to improve routine blood index abnormalities caused by lead acetate and alleviate lead acetate-induced HSC aging and aging-related inflammatory responses. Lead acetate can cause DNA damage in HSCs and induce cells to produce *γ*-H2AX. Rg1 can reduce the DNA damage-induced increases in p53 transcription and translation but does not affect the activity of P16, which results in the amelioration of lead acetate-induced HSC damage [41]. Studies also found that Rg1 can attenuate ROS production to improve HSC function in various settings [40, 42–44]. For example, Rg1 can decrease ROS production and further increase the ratio of Bcl-2/Bax in the radiation-induced HSC mitochondrial apoptosis (Figure 6).

Furthermore, Rg1 may inhibit some key genes in the p16^INK4a^-Rb, p53-p21^Cip/Waf1^, and SIRT6/NF-*κ*B signaling pathways to protect against HSC aging induced by D-gal, t-BHP, and radiation. The mechanism involves reducing DNA damage, regulating the cell cycle, adjusting telomerase activity, and compensating for the HSC telomere length [42, 45–47].

## 5. Overview of the Regulation of HPCs and Mature Blood Cells by Rg1

After HSCs differentiate into multipotent progenitors (MPPs), they develop into common myeloid progenitors (CMPs) and common lymphoid progenitors (CLPs), the classic pathway for the differentiation of HPCs [48].

Ginseng extract affects immune cell functions differently according to the specific ginsenoside profile, and the immunomodulatory effects of various ginsenoside monomers are different [49]. For example, Rg1 inhibits TNF-*α* expression in THP-1 human leukemia cells, whereas the ginsenoside Rh1 increases TNF-*α* expression [50]. An Rg1/Rb1 mixture and Rg1 exert different effects on IL-6 and TNF-*α* [49, 51]. These effects are also exhibited by the effect of total ginsenosides or Rg1 monomers on dendritic cells (DCs). Total saponins in ginseng roots can inhibit the maturation of DCs in the presence of lipopolysaccharide (LPS) [51]. However, 10 *μ*g/ml Rg1 can increase CD83, CD80, and HLA-DR expression, reduce CD14 expression in DCs derived from human peripheral blood mononuclear cells, and induce DCs to secrete cytokines (IL-6, TNF-*α*, and IL-1*β*) and chemokines (such as IL-8 and IP-10) [52]. Rg1 can stimulate the proliferation of human granulocyte-macrophage progenitors (GMPs) [53]. GMPs can develop into monocytes and myeloblasts. In LPS-activated macrophages, 10 *μ*M Rg1 can also increase the TNF-*α* levels and decrease the IL-6 protein levels, and these effects are related to regulation of the NF-*κ*B and PI3K/Akt/mTOR pathways [54]. Moreover, 50 *μ*M Rg1 can inhibit RAW264.7 macrophage apoptosis induced by serum deprivation by activating autophagy, and the AMPK/mTOR pathway is one of the signaling pathways associated with the antiapoptotic effects of Rg1 [55]. Rg1 has no obvious effect on megakaryocytes in the spleen of CY-induced mice [27] but can inhibit platelet activation by inhibiting the ERK pathway and attenuate arterial thrombosis [56]. In addition, Rg1 can reduce the infiltration of eosinophils and mast cells in a mouse model of allergic rhinitis [57].

CLPs comprise another important branch of developing HPCs that have the potential to differentiate and develop into T cells, B cells, and natural killer (NK) cells [58]. Rg1 increases the proportion of T helper (Th) cells among total T cells and increases NK cell activity in the mouse spleen [5]. Specifically, Rg1 directly enhances the Th cell response without the participation of antigen-presenting cells (APCs) by increasing the IL-4 and IL-2 levels and reducing the IFN-*γ* levels to reduce the T helper type 1 (Th1) cell population and increase the T helper type 2 (Th2) cell population in the spleen [59]. In a murine sepsis model, Rg1 increases the neutrophil count in the abdominal cavity and inhibits lymphocyte apoptosis in the thymus and spleen [60].

**Figure 7:**
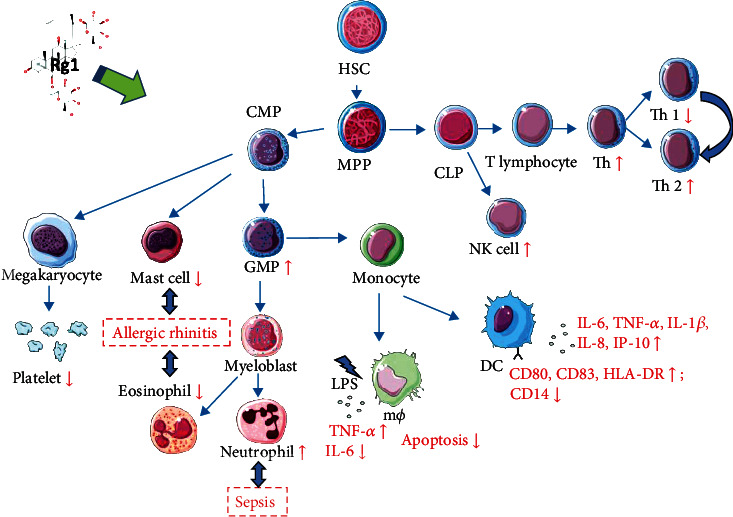
Rg1 regulates HPCs and mature blood cells in the myeloid/lymphoid cell lineage. HSC: hematopoietic stem cell; MPP: multipotent progenitor; CMP: common myeloid progenitor; GMP: granulocyte-macrophage progenitor; LPS: lipopolysaccharide; m*φ*: macrophage; DC: dendritic cell; CLP: common lymphoid progenitor; NK: natural killer cell; Th: T helper.

In total, various in vitro cell stimulation experiments and in vivo animal disease models have shown that Rg1 could regulate the development of myeloid and lymphoid progenitor cells and affect the activity and secretion of mature blood cells, and the progeny of HPCs has also been shown to regulate HPC behavior, which suggests that Rg1 may effectively regulate both innate and adaptive immunity (Figure 7).

## 6. Rg1 May Indirectly Regulate the HSC Niche

Niches with various functions exist in different areas of the bone marrow. For example, the endosteal niche can support the quiescence and maintenance of HSCs, whereas the arteriolar niche maintains quiescent HSCs, and the sinusoidal niche supports the cycling of HSCs [61, 62]. New studies have also indicated that HSCs in perisinusoidal niches are protected from aging [63]. Moreover, MSCs, the vasculature, and nerve fibers can maintain quiescent HSCs and/or control HPC differentiation through cell-to-cell communication within the niche [64]. Importantly, Rg1 may be able to regulate MSCs, endothelial cells, nerve cells, and other cells in the niche to support HSC quiescence or development.

MSCs are critical niche constituents of the bone marrow and are major contributors to many currently known niche factors, such as CXCL12, SDF, and IL-7 [65]. Rg1 can effectively regulate MSC proliferation, differentiation, senescence, and apoptosis [17]. Furthermore, studies on bone marrow MSCs in aging D-gal rats have shown that Rg1 could directly enhance the antioxidant and anti-inflammatory capabilities of bone marrow MSCs, improve the microenvironment, and further prevent HSC senescence [30, 66]. These results show that Rg1 could prevent HSC senescence by regulating MSCs in the bone marrow niche.

Vascular endothelial cells play roles in supporting the transport of HSCs [67], and endothelial-related signals (e.g., Notch ligands and E-selectin) might regulate HSC expansion and bone marrow hematopoiesis after myelosuppressive stress [68]. Rg1 can induce vascular endothelial growth factor expression in human endothelial cells and promote proliferation, migration, adhesion, and vasculogenesis in vitro [69, 70]. These results indicate that Rg1 may expand HSCs by regulating endothelial cells in the HSC niche.

HSCs mostly exist in a state of quiescence, and alterations in the metabolism of quiescent HSCs help these cells survive for extended periods of time in hypoxic environments [71]. The stimulation of quiescent HSCs by cell damage initiates active division. The dysregulation of these transitions can lead to stem cell exhaustion or the gradual loss of active HSCs. Studies have shown that the adrenergic nerves of the sympathetic nervous system mobilize HSCs and promote the recovery of hematopoietic function in the niche; moreover, adrenergic nerve-related Schwann cells may contribute to the quiescence of HSCs through TGF-*β* signaling [72, 73]. Rg1 promotes the proliferation of primary Schwann cells and the expression of neurotrophic factors while supporting the resistance of these cells to hydrogen peroxide-induced oxidative damage [74, 75]. This finding suggests that Rg1 may maintain quiescent HSCs by regulating Schwann cells.

Bone marrow adipocytes can act as negative regulators of the hematopoietic microenvironment [76]. Rg1 inhibits the development and maturation of adipocytes by activating C/EBP homologous protein 10 in 3T3-L1 cells [77]. This finding suggests that Rg1 may also antagonize bone marrow adipogenesis and thereby benefits the hematopoietic microenvironment and protects HSCs.

**Figure 8:**
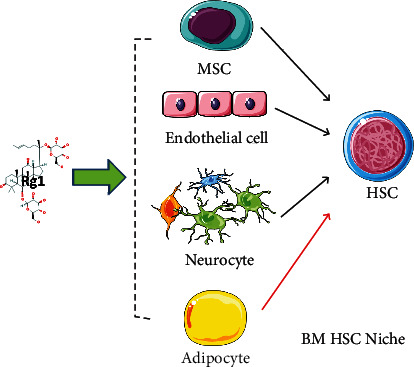
Rg1 may regulate MSCs, endothelial cells, neurocytes, and adipocytes in the bone marrow niche and thus indirectly maintain HSCs.

In summary, in the bone marrow niche, Rg1 may alleviate HSC senescence through MSCs, regulate endothelial cells to expand HSCs, activate Schwann cells to maintain quiescent HSCs, and protect HSCs by inhibiting the formation of adipocytes (Figure 8). However, the relevant direct evidence must be verified by experimental data.

## 7. Conclusions and Remarks

Through the use of bioinformatics and molecular docking methods to analyze the molecular pharmacological mechanism through which Rg1 regulates HSCs/HPCs, we predicted that GVHD is a possible disease target of Rg1 therapy and that ACE is a potential target protein through which Rg1 regulates the proliferation and differentiation of HSCs/HPCs. A review of the literature also showed that Rg1 may regulate HSC proliferation and can activate extramedullary HSCs to migrate to the bone marrow. These results suggest a new strategy for HSC expansion in vitro and a new method for improving the HSC homing rate and alleviating GHVD in HSC transplantation in vivo. Moreover, the ability of Rg1 to alleviate HSC aging and regulate HPC development suggests that Rg1 exerts direct effects on the maintenance of HSCs/HPCs. However, whether Rg1 can promote the proliferation of HSCs without affecting their differentiation in vitro and whether Rg1 can enhance the HSC homing rate while reducing GVHD during HSC transplantation in vivo are worth further comprehensive exploration.

## Supplementary Materials

Supplementary Materials Supplementary Material 1: putative targets of Rg1 identified using TargetNet. Supplementary Material 2: putative targets of Rg1 identified using SwissTargetPrediction. Supplementary Material 3: genes involved in HSC proliferation. Supplementary Material 4: genes involved in HSC migration. Supplementary Material 5: genes involved in HSC differentiation. Supplementary Material 6: genes involved in HPC differentiation. Supplementary Material 7: databases used in this study.

## References

[1] Zhao S. J., Hou C. X., Xu L. X., Liang Y. L., Qian Y. C., Sun Y. (2011). Effects of suppressing oleanane-type ginsenoside biosynthesis on dammarane-type ginsenoside production. *Journal of Jilin University (Engineering and Technology Edition)*.

[2] Han J. Y., Kim M. J., Ban Y. W., Hwang H. S., Choi Y. E. (2013). The involvement of -amyrin 28-oxidase (CYP716A52v2) in oleanane-type ginsenoside biosynthesis in Panax ginseng. *Plant & Cell Physiology*.

[3] Chen X. C., Chen Y., Zhu Y. G., Fang F., Chen L. M. (2011). Protective effect of ginsenoside Rg1 against MPTP-induced apoptosis in mouse substantia nigra neurons. *Acta Pharmacologica Sinica*.

[4] Li L., Pan C. S., Yan L. (2018). Ginsenoside Rg1 ameliorates rat myocardial ischemia-reperfusion injury by modulating energy metabolism pathways. *Frontiers in Physiology*.

[5] Kenarova B., Neychev H., Hadjiivanova C., Petkov V. D. (1990). Immunomodulating activity of ginsenoside Rg_1_ from *Panax ginseng*. *Japanese Journal of Pharmacology*.

[6] Tian J., Shi J., Wei M. (2016). The efficacy and safety of Fufangdanshen tablets (Radix Salviae miltiorrhizae formula tablets) for mild to moderate vascular dementia: a study protocol for a randomized controlled trial. *Trials*.

[7] Cho S. K., Kim D., Yoo D., Jang E. J., Jun J. B., Sung Y. K. (2018). Korean Red Ginseng exhibits no significant adverse effect on disease activity in patients with rheumatoid arthritis: a randomized, double-blind, crossover study. *Journal of Ginseng Research*.

[8] Dzierzak E., Bigas A. (2018). Blood development: hematopoietic stem cell dependence and independence. *Cell Stem Cell*.

[9] Wilson A., Laurenti E., Oser G. (2008). Hematopoietic stem cells reversibly switch from dormancy to self-renewal during homeostasis and repair. *Cell*.

[10] Kovtonyuk L. V., Fritsch K., Feng X., Manz M. G., Takizawa H. (2016). Inflamm-aging of hematopoiesis, hematopoietic stem cells, and the bone marrow microenvironment. *Frontiers in Immunology*.

[11] Birbrair A., Frenette P. S. (2016). Niche heterogeneity in the bone marrow. *Annals of the New York Academy of Sciences*.

[12] Pei X. (1999). Who is hematopoietic stem cell: CD34+ or CD34-?. *International Journal of Hematology*.

[13] Matsuoka S., Ebihara Y., Xu M. (2001). CD34 expression on long-term repopulating hematopoietic stem cells changes during developmental stages. *Blood*.

[14] Chen J., Ellison F. M., Keyvanfar K. (2008). Enrichment of hematopoietic stem cells with SLAM and LSK markers for the detection of hematopoietic stem cell function in normal and Trp53 null mice. *Experimental Hematology*.

[15] Challen G. A., Boles N., Lin K. K., Goodell M. A. (2009). Mouse hematopoietic stem cell identification and analysis. *Cytometry. Part A*.

[16] Czechowicz A., Palchaudhuri R., Scheck A. (2019). Selective hematopoietic stem cell ablation using CD117-antibody-drug- conjugates enables safe and effective transplantation with immunity preservation. *Nature Communications*.

[17] He F., Yu C., Liu T., Jia H. (2020). Ginsenoside Rg1 as an effective regulator of mesenchymal stem cells. *Frontiers in Pharmacology*.

[18] Yao Z. J., Dong J., Che Y. J. (2016). TargetNet: a web service for predicting potential drug-target interaction profiling via multi-target SAR models. *Journal of Computer-Aided Molecular Design*.

[19] Daina A., Michielin O., Zoete V. (2019). SwissTargetPrediction: updated data and new features for efficient prediction of protein targets of small molecules. *Nucleic Acids Research*.

[20] Zhou Y., Zhou B., Pache L. (2019). Metascape provides a biologist-oriented resource for the analysis of systems-level datasets. *Nature Communications*.

[21] Bardou P., Mariette J., Escudié F., Djemiel C., Klopp C. (2014). jvenn: an interactive Venn diagram viewer. *BMC Bioinformatics*.

[22] Itkin T., Gur-Cohen S., Spencer J. A. (2016). Distinct bone marrow blood vessels differentially regulate haematopoiesis. *Nature*.

[23] Xu S. F., Yu L. M., Fan Z. H. (2012). Improvement of ginsenoside Rg1 on hematopoietic function in cyclophosphamide- induced myelosuppression mice. *European Journal of Pharmacology*.

[24] Fraiser L. H., Kanekal S., Kehrer J. P. (1991). Cyclophosphamide toxicity. *Drugs*.

[25] Wang Y., Meng Q., Qiao H., Jiang H., Sun X. (2009). Role of the spleen in cyclophosphamide-induced hematosuppression and extramedullary hematopoiesis in mice. *Archives of Medical Research*.

[26] O'Malley D. P., Kim Y. S., Perkins S. L., Baldridge L. A., Juliar B. E., Orazi A. (2005). Morphologic and immunohistochemical evaluation of splenic hematopoietic proliferations in neoplastic and benign disorders. *Modern Pathology*.

[27] Liu H. H., Chen F. P., Liu R. K., Lin C. L., Chang K. T. (2015). Ginsenoside Rg1 improves bone marrow haematopoietic activity via extramedullary haematopoiesis of the spleen. *Journal of Cellular and Molecular Medicine*.

[28] Wilson A., Trumpp A. (2006). Bone-marrow haematopoietic-stem-cell niches. *Nature Reviews Immunology*.

[29] Krause D. S., Fackler M. J., Civin C. I., May W. S. (1996). CD34: structure, biology, and clinical utility [see comments]. *Blood*.

[30] Hu W., Jing P., Wang L., Zhang Y., Yong J., Wang Y. (2015). The positive effects of ginsenoside Rg1 upon the hematopoietic microenvironment in a D-galactose-induced aged rat model. *BMC Complementary and Alternative Medicine*.

[31] Sharma M., Afrin F., Satija N., Tripathi R. P., Gangenahalli G. U. (2011). Stromal-derived factor-1/CXCR4 signaling: indispensable role in homing and engraftment of hematopoietic stem cells in bone marrow. *Stem Cells and Development*.

[32] Hu A., Shuai Z., Liu J. (2020). Ginsenoside Rg1 prevents vascular intimal hyperplasia involved by SDF-1*α*/CXCR4, SCF/c-kit and FKN/CX3CR1 axes in a rat balloon injury. *Journal of Ethnopharmacology*.

[33] De Haan G., Lazare S. S. (2018). Aging of hematopoietic stem cells. *Blood*.

[34] Moehrle B. M., Geiger H. (2016). Aging of hematopoietic stem cells: DNA damage and mutations?. *Experimental Hematology*.

[35] Ou H. L., Schumacher B. (2018). DNA damage responses and p53 in the aging process. *Blood*.

[36] Wei H., Li L., Song Q. (2005). Behavioural study of the d-galactose induced aging model in C57BL/6J mice. *Behavioural Brain Research*.

[37] Cui X., Zuo P., Zhang Q. (2006). Chronic systemic D-galactose exposure induces memory loss, neurodegeneration, and oxidative damage in mice: protective effects of R-*α*-lipoic acid. *Journal of Neuroscience Research*.

[38] Scheller E. L., Chang J., Wang C. Y. (2008). Wnt/*β*-catenin inhibits dental pulp stem cell differentiation. *Journal of Dental Research*.

[39] Kühl S. J., Kühl M. (2013). On the role of Wnt/*β*-catenin signaling in stem cells. *Biochimica et Biophysica Acta*.

[40] Li J., Cai D., Yao X. (2016). Protective effect of ginsenoside rg1 on hematopoietic stem/progenitor cells through attenuating oxidative stress and the Wnt/*β*-catenin signaling pathway in a mouse model of d-galactose-induced aging. *International Journal of Molecular Sciences*.

[41] Cai S. Z., Zhou Y., Liu J. (2018). Alleviation of ginsenoside Rg1 on hematopoietic homeostasis defects caused by lead-acetate. *Biomedicine & Pharmacotherapy*.

[42] Chen C., Mu X. Y., Zhou Y. (2014). Ginsenoside Rg1 enhances the resistance of hematopoietic stem/progenitor cells to radiation-induced aging in mice. *Acta Pharmacologica Sinica*.

[43] Shao L., Lou Y., Zhou D. (2013). Hematopoietic stem cell injury induced by ionizing radiation. *Antioxidants & Redox Signaling*.

[44] Cao H., Wei W., Xu R., Cui X. (2021). Ginsenoside Rg1 can restore hematopoietic function by inhibiting Bax translocation-mediated mitochondrial apoptosis in aplastic anemia. *Scientific Reports*.

[45] Yue Z., Rong J., Ping W. (2014). Gene expression of the p16INK4a-Rb and p19Arf-p53-p21Cip/Waf1 signaling pathways in the regulation of hematopoietic stem cell aging by ginsenoside Rg1. *Genetics and Molecular Research*.

[46] Tang Y. L., Zhou Y., Wang Y. P., Wang J. W., Ding J. C. (2015). SIRT6/NF-*κ*B signaling axis in ginsenoside Rg1-delayed hematopoietic stem/progenitor cell senescence. *International Journal of Clinical and Experimental Pathology*.

[47] Zhou Y., Liu J., Cai S., Liu D., Jiang R., Wang Y. (2015). Protective effects of ginsenoside Rg1 on aging Sca-1^+^ hematopoietic cells. *Molecular Medicine Reports*.

[48] Zhang Y., Gao S., Xia J., Liu F. (2018). Hematopoietic hierarchy - an updated roadmap. *Trends in Cell Biology*.

[49] Attele A. S., Wu J. A., Yuan C. S. (1999). Ginseng pharmacology: multiple constituents and multiple actions. *Biochemical Pharmacology*.

[50] Wang Y., Wang B. X., Liu T. H., Minami M., Nagata T., Ikejima T. (2000). Metabolism of ginsenoside Rg1 by intestinal bacteria. II. Immunological activity of ginsenoside Rg1 and Rh1. *Acta Pharmacologica Sinica*.

[51] Lee Y. J., Son Y. M., Gu M. J. (2015). Ginsenoside fractions regulate the action of monocytes and their differentiation into dendritic cells. *Journal of Ginseng Research*.

[52] Huang Y., Zou Y., Lin L., Zheng R. (2017). Ginsenoside Rg1 activates dendritic cells and acts as a vaccine adjuvant inducing protective cellular responses against lymphomas. *DNA and Cell Biology*.

[53] Niu Y. P., Jin J. M., Gao R. L., Xie G. L., Chen X. H. (2001). Effects of ginsenosides Rg1 and Rb1 on proliferation of human marrow granulocyte-macrophage progenitor cells. *Zhongguo Shi Yan Xue Ye Xue Za Zhi*.

[54] Wang Y., Liu Y., Zhang X. Y. (2014). Ginsenoside Rg1 regulates innate immune responses in macrophages through differentially modulating the NF-*κ*B and PI3K/Akt/mTOR pathways. *International Immunopharmacology*.

[55] Yang P., Ling L., Sun W. (2018). Ginsenoside Rg1 inhibits apoptosis by increasing autophagy via the AMPK/mTOR signaling in serum deprivation macrophages. *Acta Biochimica et Biophysica Sinica*.

[56] Zhou Q., Jiang L., Xu C. (2014). Ginsenoside Rg1 inhibits platelet activation and arterial thrombosis. *Thrombosis Research*.

[57] Oh H. A., Seo J. Y., Jeong H. J., Kim H. M. (2013). Ginsenoside Rg1 inhibits the TSLP production in allergic rhinitis mice. *Immunopharmacology and Immunotoxicology*.

[58] Kondo M. (2016). One niche to rule both maintenance and loss of stemness in HSCs. *Immunity*.

[59] Lee E. J., Ko E., Lee J. (2004). Ginsenoside Rg1 enhances CD4^+^ T-cell activities and modulates Th1/Th2 differentiation. *International Immunopharmacology*.

[60] Zou Y., Tao T., Tian Y. (2013). Ginsenoside Rg1 improves survival in a murine model of polymicrobial sepsis by suppressing the inflammatory response and apoptosis of lymphocytes. *The Journal of Surgical Research*.

[61] Nakamura Y., Arai F., Iwasaki H. (2010). Isolation and characterization of endosteal niche cell populations that regulate hematopoietic stem cells. *Blood*.

[62] Silberstein L. E., Lin C. P. (2013). A new image of the hematopoietic stem cell vascular niche. *Cell Stem Cell*.

[63] Kunisaki Y., Bruns I., Scheiermann C. (2013). Arteriolar niches maintain haematopoietic stem cell quiescence. *Nature*.

[64] Wei Q., Frenette P. S. (2018). Niches for hematopoietic stem cells and their progeny. *Immunity*.

[65] Cordeiro Gomes A., Hara T., Lim V. Y. (2016). Hematopoietic stem cell niches produce lineage-instructive signals to control multipotent progenitor differentiation. *Immunity*.

[66] Zeng Y., Hu W., Jing P. (2018). The regulation of ginsenoside Rg1 upon aging of bone marrow stromal cell contribute to delaying senescence of bone marrow mononuclear cells (BMNCs). *Life Sciences*.

[67] Ramasamy S. K., Kusumbe A. P., Itkin T., Gur-Cohen S., Lapidot T., Adams R. H. (2016). Regulation of hematopoiesis and osteogenesis by blood vessel-derived signals. *Annual Review of Cell and Developmental Biology*.

[68] Winkler I. G., Barbier V., Nowlan B. (2012). Vascular niche E-selectin regulates hematopoietic stem cell dormancy, self renewal and chemoresistance. *Nature Medicine*.

[69] Leung K. W., Pon Y. L., Wong R. N., Wong A. S. (2006). Ginsenoside-Rg1 induces vascular endothelial growth factor expression through the glucocorticoid receptor-related phosphatidylinositol 3-kinase/Akt and *β*-catenin/T-cell factor-dependent pathway in human endothelial cells∗. *The Journal of Biological Chemistry*.

[70] Shi A. W., Wang X. B., Lu F. X., Zhu M. M., Kong X. Q., Cao K. J. (2009). Ginsenoside Rg1 promotes endothelial progenitor cell migration and proliferation. *Acta Pharmacologica Sinica*.

[71] Srikanth L., Sunitha M. M., Venkatesh K. (2015). Anaerobic glycolysis and HIF1*α* expression in haematopoietic stem cells explains its quiescence nature. *Journal of Stem Cells*.

[72] Méndez-Ferrer S., Michurina T. V., Ferraro F. (2010). Mesenchymal and haematopoietic stem cells form a unique bone marrow niche. *Nature*.

[73] Yamazaki S., Ema H., Karlsson G. (2011). Nonmyelinating Schwann cells maintain hematopoietic stem cell hibernation in the bone marrow niche. *Cell*.

[74] Liang W., Ge S., Yang L. (2010). Ginsenosides Rb1 and Rg1 promote proliferation and expression of neurotrophic factors in primary Schwann cell cultures. *Brain Research*.

[75] Ma J., Liu J., Wang Q., Yu H., Chen Y., Xiang L. (2013). The beneficial effect of ginsenoside Rg1 on Schwann cells subjected to hydrogen peroxide induced oxidative injury. *International Journal of Biological Sciences*.

[76] Naveiras O., Nardi V., Wenzel P. L., Hauschka P. V., Fahey F., Daley G. Q. (2009). Bone-marrow adipocytes as negative regulators of the haematopoietic microenvironment. *Nature*.

[77] Koh E. J., Kim K. J., Choi J., Jeon H. J., Seo M. J., Lee B. Y. (2017). Ginsenoside Rg1 suppresses early stage of adipocyte development via activation of C/EBP homologous protein-10 in 3T3-L1 and attenuates fat accumulation in high fat diet-induced obese zebrafish. *Journal of Ginseng Research*.

