# Supplementary material for: Ginsenoside Rg1 as a Potential Regulator of Hematopoietic Stem/Progenitor Cells
Source: Stem Cells Int. 2021 Dec 31;2021:4633270. doi: 10.1155/2021/4633270 (PMC8741398; doi:10.1155/2021/4633270)
Supplement: Supplementary Materials — Supplementary Material 1: putative targets of Rg1 identified using TargetNet. Supplementary Material 2: putative targets of Rg1 identified using SwissTargetPrediction. Supplementary Material 3: genes involved in HSC proliferation. Supplementary Material 4: genes involved in HSC migration. Supplementary Material 5: genes involved in HSC differentiation. Supplementary Material 6: genes involved in HPC differentiation. Supplementary Material 7: databases used in this study. [file 4633270.f1.zip › Supplementary Material 1.pdf]

Uniprot_ID	Protein	Prob
P31639	Sodium/glucose cotransporter 2	1
P13866	Sodium/glucose cotransporter 1	1
P17706	Tyrosine-protein phosphatase non-receptor type 2	0.999
P18031	Tyrosine-protein phosphatase non-receptor type 1	0.994
O42713	Polyphenol oxidase 2	0.968
P15207	Androgen receptor	0.886
P29477	Nitric oxide synthase, inducible	0.745
P47936	Cannabinoid receptor 2	0.682
P26358	DNA (cytosine-5)-methyltransferase 1	0.452
P04058	Acetylcholinesterase	0.354
P24468	COUP transcription factor 2	0.225
P08483	Muscarinic acetylcholine receptor M3	0.102
P80365	Corticosteroid 11-beta-dehydrogenase isozyme 2	0.067
Q06418	Tyrosine-protein kinase receptor TYRO3	0.018
P30305	M-phase inducer phosphatase 2	0.014
Q9H228	Sphingosine 1-phosphate receptor 5	0.01
P10275	Androgen receptor	0.001
P07900	Heat shock protein HSP 90-alpha	0.001
P31749	RAC-alpha serine/threonine-protein kinase	0
P34998	Corticotropin-releasing factor receptor 1	0
P31213	3-oxo-5-alpha-steroid 4-dehydrogenase 2	0
P34995	Prostaglandin E2 receptor EP1 subtype	0
Q9BY41	Histone deacetylase 8	0
P08575	Receptor-type tyrosine-protein phosphatase C	0
P03372	Estrogen receptor	0
P08473	Neprilysin	0
P20272	Cannabinoid receptor 1	0
Q14524	Sodium channel protein type 5 subunit alpha	0
P08183	Multidrug resistance protein 1	0
P18130	Alpha-1A adrenergic receptor	0
P79208	Prostaglandin G/H synthase 2	0
O43614	Orexin receptor type 2	0
Q05397	Focal adhesion kinase 1	0
P00766	Chymotrypsinogen A	0
O35433	Transient receptor potential cation channel subfamily V member 1	0
P00760	Cationic trypsin	0
Q9Y2R2	Tyrosine-protein phosphatase non-receptor type 22	0
P05177	Cytochrome P450 1A2	0
P51677	C-C chemokine receptor type 3	0
P07858	Cathepsin B	0
Q9Y5Y4	Prostaglandin D2 receptor 2	0
Q9H244	P2Y purinoceptor 12	0
P30291	Wee1-like protein kinase	0
P29089	Type-1B angiotensin II receptor	0
P33765		0
Q07422	Bifunctional dihydrofolate reductase-thymidylate synthase	0
P25104	Type-1 angiotensin II receptor	0
P25105	Platelet-activating factor receptor	0
P63001	Ras-related C3 botulinum toxin substrate 1	0
P14600	Substance-P receptor	0
P25100	Alpha-1D adrenergic receptor	0

P05622	Platelet-derived growth factor receptor beta	0
Q14416	Metabotropic glutamate receptor 2	0
P08842	Steryl-sulfatase	0
P16116	Aldose reductase	0
P10827	Thyroid hormone receptor alpha	0
P10826	Retinoic acid receptor beta	0
Q13085	Acetyl-CoA carboxylase 1	0
P10828	Thyroid hormone receptor beta	0
P10276	Retinoic acid receptor alpha	0
P15121	Aldose reductase	0
P56658	Adenosine deaminase	0
P19320	Vascular cell adhesion protein 1	0
Q96EB6	NAD-dependent protein deacetylase sirtuin-1	0
Q07912	Activated CDC42 kinase 1	0
P19327	5-hydroxytryptamine receptor 1A	0
O00767	Acyl-CoA desaturase	0
O43193	Motilin receptor	0
O00763	Acetyl-CoA carboxylase 2	0
O00408	cGMP-dependent 3',5'-cyclic phosphodiesterase	0
Q9UM73	ALK tyrosine kinase receptor	0
Q6B856	Tubulin beta-2B chain	0
Q01727	Melanocyte-stimulating hormone receptor	0
Q01726	Melanocyte-stimulating hormone receptor	0
P18084	Integrin beta-5	0
P08253	72 kDa type IV collagenase	0
P25929	Neuropeptide Y receptor type 1	0
P18089	Alpha-2B adrenergic receptor	0
P35368	Alpha-1B adrenergic receptor	0
P35367	Histamine H1 receptor	0
Q15722	Leukotriene B4 receptor 1	0
P09917	Arachidonate 5-lipoxygenase	0
P21451	Endothelin B receptor	0
P21452	Substance-K receptor	0
P21453	Sphingosine 1-phosphate receptor 1	0
Q07820	Induced myeloid leukemia cell differentiation protein Mcl-1	0
Q494W8	CHRNA7-FAM7A fusion protein	0
Q02156	Protein kinase C epsilon type	0
Q8TDS4	Hydroxycarboxylic acid receptor 2	0
P36544	Neuronal acetylcholine receptor subunit alpha-7	0
Q04631	Protein farnesyltransferase/geranylgeranyltransferase type-1 subunit alpha	0
P12268	Inosine-5'-monophosphate dehydrogenase 2	0
P50052	Type-2 angiotensin II receptor	0
P19099	Cytochrome P450 11B2, mitochondrial	0
P43140	Alpha-1A adrenergic receptor	0
P49286	Melatonin receptor type 1B	0
P13497	Bone morphogenetic protein 1	0
P04035	3-hydroxy-3-methylglutaryl-coenzyme A reductase	0
P46098	5-hydroxytryptamine receptor 3A	0
P06401	Progesterone receptor	0
P51452	Dual specificity protein phosphatase 3	0
P35563	5-hydroxytryptamine receptor 3A	0

O14672	Disintegrin and metalloproteinase domain-containing protein 10	0
Q9P1W9	Serine/threonine-protein kinase pim-2	0
P23795	Acetylcholinesterase	0
Q92847	Growth hormone secretagogue receptor type 1	0
P50579	Methionine aminopeptidase 2	0
P37268	Squalene synthase	0
P09960	Leukotriene A-4 hydrolase	0
P30542	Adenosine receptor A1	0
P30543	Adenosine receptor A2a	0
P23944	Alpha-1D adrenergic receptor	0
Q9R0C9	Sigma non-opioid intracellular receptor 1	0
P25774	Cathepsin S	0
P25779	Cruzipain	0
Q5S007	Leucine-rich repeat serine/threonine-protein kinase 2	0
P30729	D(4) dopamine receptor	0
P34972	Cannabinoid receptor 2	0
P37288	Vasopressin V1a receptor	0
Q04759	Protein kinase C theta type	0
P23458	Tyrosine-protein kinase JAK1	0
P25025	C-X-C chemokine receptor type 2	0
P25024	C-X-C chemokine receptor type 1	0
P41968	Melanocortin receptor 3	0
P25021	Histamine H2 receptor	0
P12822	Angiotensin-converting enzyme	0
P12821	Angiotensin-converting enzyme	0
P04049	RAF proto-oncogene serine/threonine-protein kinase	0
P00750	Tissue-type plasminogen activator	0
P43119	Prostacyclin receptor	0
Q02750	Dual specificity mitogen-activated protein kinase kinase 1	0
P32240	Prostaglandin E2 receptor EP4 subtype	0
P52333	Tyrosine-protein kinase JAK3	0
Q04206	Transcription factor p65	0
P49759	Dual specificity protein kinase CLK1	0
P55245	Epidermal growth factor receptor	0
P04626	Receptor tyrosine-protein kinase erbB-2	0
P11802	Cyclin-dependent kinase 4	0
P04629	High affinity nerve growth factor receptor	0
P54760	Ephrin type-B receptor 4	0
P05106	Integrin beta-3	0
Q13946	High affinity cAMP-specific 3',5'-cyclic phosphodiesterase 7A	0
P51679	C-C chemokine receptor type 4	0
P56817	Beta-secretase 1	0
Q12809	Potassium voltage-gated channel subfamily H member 2	0
Q00535	Cyclin-dependent-like kinase 5	0
P13631	Retinoic acid receptor gamma	0
O43497	Voltage-dependent T-type calcium channel subunit alpha-1G	0
P25099	Adenosine receptor A1	0
P51639	3-hydroxy-3-methylglutaryl-coenzyme A reductase	0
P11362	Fibroblast growth factor receptor 1	0
P41144	Kappa-type opioid receptor	0

P41145	Kappa-type opioid receptor	0
P41146	Nociceptin receptor	0
Q15761	Neuropeptide Y receptor type 5	0
P41143	Delta-type opioid receptor	0
P09619	Platelet-derived growth factor receptor beta	0
Q8IXJ6	NAD-dependent protein deacetylase sirtuin-2	0
P33032	Melanocortin receptor 5	0
P21918	D(1B) dopamine receptor	0
P21917	D(4) dopamine receptor	0
P78396	Cyclin-A1	0
P42866	Mu-type opioid receptor	0
P43088	Prostaglandin F2-alpha receptor	0
P48147	Prolyl endopeptidase	0
P48145	Neuropeptides B/W receptor type 1	0
P14740	Dipeptidyl peptidase 4	0
P21980	Protein-glutamine gamma-glutamyltransferase 2	0
P35610	Sterol O-acyltransferase 1	0
P42345	Serine/threonine-protein kinase mTOR	0
Q9Y243	RAC-gamma serine/threonine-protein kinase	0
Q00788	Vasopressin V2 receptor	0
P10980	Muscarinic acetylcholine receptor M2	0
P11511	Aromatase	0
P08069	Insulin-like growth factor 1 receptor	0
P23385	Metabotropic glutamate receptor 1	0
P35354	Prostaglandin G/H synthase 2	0
P35353	Corticotropin-releasing factor receptor 1	0
P35218	Carbonic anhydrase 5A, mitochondrial	0
O60725	Protein-S-isoprenylcysteine O-methyltransferase	0
P22460	Potassium voltage-gated channel subfamily A member 5	0
Q29463	Anionic trypsin	0
P05362	Intercellular adhesion molecule 1	0
P62993	Growth factor receptor-bound protein 2	0
P05364	Beta-lactamase	0
P48736	Phosphatidylinositol 4,5-bisphosphate 3-kinase catalytic subunit gamma isoform	0
Q02127	Dihydroorotate dehydrogenase (quinone), mitochondrial	0
P00374	Dihydrofolate reductase	0
P00375	Dihydrofolate reductase	0
P16234	Platelet-derived growth factor receptor alpha	0
P28702	Retinoic acid receptor RXR-beta	0
P43405	Tyrosine-protein kinase SYK	0
P08588	Beta-1 adrenergic receptor	0
P43403	Tyrosine-protein kinase ZAP-70	0
Q00960	Glutamate receptor ionotropic, NMDA 2B	0
Q92731	Estrogen receptor beta	0
P08581	Hepatocyte growth factor receptor	0
Q920D2	Dihydrofolate reductase	0
P80457	Xanthine dehydrogenase/oxidase	0
P07711	Cathepsin L1	0
Q03181	Peroxisome proliferator-activated receptor delta	0
P49137	MAP kinase-activated protein kinase 2	0
P40238	Thrombopoietin receptor	0
P34913	Bifunctional epoxide hydrolase 2	0
Q06187	Tyrosine-protein kinase BTK	0

P20288	D(2) dopamine receptor	0
Q13639	5-hydroxytryptamine receptor 4	0
P36897	TGF-beta receptor type-1	0
P01375	Tumor necrosis factor	0
Q8NER1	Transient receptor potential cation channel subfamily V member 1	0
P30518	Vasopressin V2 receptor	0
P05067	Amyloid beta A4 protein	0
P27338	Amine oxidase [flavin-containing] B	0
P25103	Substance-P receptor	0
Q99705	Melanin-concentrating hormone receptor 1	0
Q62053	Prostaglandin E2 receptor EP2 subtype	0
P08311	Cathepsin G	0
Q9Y5Z0	Beta-secretase 2	0
Q95323	Carbonic anhydrase 4	0
P25101	Endothelin-1 receptor	0
Q13258	Prostaglandin D2 receptor	0
P29371	Neuromedin-K receptor	0
P25116	Proteinase-activated receptor 1	0
P26684	Endothelin-1 receptor	0
P30939	5-hydroxytryptamine receptor 1F	0
P08912	Muscarinic acetylcholine receptor M5	0
P08913	Alpha-2A adrenergic receptor	0
O43613	Orexin receptor type 1	0
P49810	Presenilin-2	0
P29274	Adenosine receptor A2a	0
P29275	Adenosine receptor A2b	0
Q9QZN9	Cannabinoid receptor 2	0
P19643	Amine oxidase [flavin-containing] B	0
P41180	Extracellular calcium-sensing receptor	0
P31390	Histamine H1 receptor	0
P31391	Somatostatin receptor type 4	0
Q14790	Caspase-8	0
Q02769	Squalene synthase	0
Q15077	P2Y purinoceptor 6	0
O43570	Carbonic anhydrase 12	0
Q13464	Rho-associated protein kinase 1	0
Q02763	Angiopoietin-1 receptor	0
Q15078	Cyclin-dependent kinase 5 activator 1	0
P24941	Cyclin-dependent kinase 2	0
P49355	Protein farnesyltransferase subunit beta	0
Q62758	5-hydroxytryptamine receptor 4	0
P49356	Protein farnesyltransferase subunit beta	0
P05093	Steroid 17-alpha-hydroxylase/17,20 lyase	0
Q9ULX7	Carbonic anhydrase 14	0
P07478	Trypsin-2	0
P29476	Nitric oxide synthase, brain	0
P29474	Nitric oxide synthase, endothelial	0
P29475	Nitric oxide synthase, brain	0
P07477	Trypsin-1	0
P21397	Amine oxidase [flavin-containing] A	0
P21396	Amine oxidase [flavin-containing] A	0
P61169	D(2) dopamine receptor	0
P28221	5-hydroxytryptamine receptor 1D	0

P28222	5-hydroxytryptamine receptor 1B	0
P28223	5-hydroxytryptamine receptor 2A	0
P08631	Tyrosine-protein kinase HCK	0
Q15759	Mitogen-activated protein kinase 11	0
P29597	Non-receptor tyrosine-protein kinase TYK2	0
P19634	Sodium/hydrogen exchanger 1	0
P35408	Prostaglandin E2 receptor EP4 subtype	0
P41149	Melanocortin receptor 5	0
Q08881	Tyrosine-protein kinase ITK/TSK	0
P00533	Epidermal growth factor receptor	0
P19793	Retinoic acid receptor RXR-alpha	0
P07333	Macrophage colony-stimulating factor 1 receptor	0
P07339	Cathepsin D	0
P05979	Prostaglandin G/H synthase 1	0
P21728	D(1A) dopamine receptor	0
P14416	D(2) dopamine receptor	0
P08709	Coagulation factor VII	0
P14555	Phospholipase A2, membrane associated	0
P28647	Adenosine receptor A3	0
O42275	Acetylcholinesterase	0
P47900	P2Y purinoceptor 1	0
P35968	Vascular endothelial growth factor receptor 2	0
P24530	Endothelin B receptor	0
P35557	Glucokinase	0
P08684	Cytochrome P450 3A4	0
Q9HC16	DNA dC->dU-editing enzyme APOBEC-3G	0
P23219	Prostaglandin G/H synthase 1	0
P35228	Nitric oxide synthase, inducible	0
P52732	Kinesin-like protein KIF11	0
O60674	Tyrosine-protein kinase JAK2	0
Q16790	Carbonic anhydrase 9	0
P30559	Oxytocin receptor	0
P30411	B2 bradykinin receptor	0
Q99500	Sphingosine 1-phosphate receptor 3	0
P30553	Gastrin/cholecystokinin type B receptor	0
P30551	Cholecystokinin receptor type A	0
P30557	Prostaglandin E2 receptor EP3 subtype	0
P30556	Type-1 angiotensin II receptor	0
P70536	Oxytocin receptor	0
P42892	Endothelin-converting enzyme 1	0
P98170	E3 ubiquitin-protein ligase XIAP	0
P56450	Melanocortin receptor 4	0
P20444	Protein kinase C alpha type	0
P05556	Integrin beta-1	0
P14174	Macrophage migration inhibitory factor	0
P06737	Glycogen phosphorylase, liver form	0
P53350	Serine/threonine-protein kinase PLK1	0
P45984	Mitogen-activated protein kinase 9	0
P53609	Geranylgeranyl transferase type-1 subunit beta	0
Q13133	Oxysterols receptor LXR-alpha	0
P45983	Mitogen-activated protein kinase 8	0
P04054	Phospholipase A2	0
Q04609	Glutamate carboxypeptidase 2	0
P08235	Mineralocorticoid receptor	0

P23443	Ribosomal protein S6 kinase beta-1	0
P00747	Plasminogen	0
P16050	Arachidonate 15-lipoxygenase	0
P00742	Coagulation factor X	0
Q5RAG0	Histone deacetylase 1	0
P00749	Urokinase-type plasminogen activator	0
Q9GZU7	Carboxy-terminal domain RNA polymerase II polypeptide A small phosphatase 1	0
P28566	5-hydroxytryptamine receptor 1E	0
P12931	Proto-oncogene tyrosine-protein kinase Src	0
P28564	5-hydroxytryptamine receptor 1B	0
P41279	Mitogen-activated protein kinase kinase kinase 8	0
P20701	Integrin alpha-L	0
P47901	Vasopressin V1b receptor	0
Q7TMR0	Lysosomal Pro-X carboxypeptidase	0
P22001	Potassium voltage-gated channel subfamily A member 3	0
Q00G26	Perilipin-5	0
P49682	C-X-C chemokine receptor type 3	0
P49841	Glycogen synthase kinase-3 beta	0
P49840	Glycogen synthase kinase-3 alpha	0
P97612	Fatty-acid amide hydrolase 1	0
Q9QYN8	Histamine H3 receptor	0
P35869	Aryl hydrocarbon receptor	0
P23141	Liver carboxylesterase 1	0
O14939	Phospholipase D2	0
P48039	Melatonin receptor type 1A	0
P31941	DNA dC->dU-editing enzyme APOBEC-3A	0
Q8TDV5	Glucose-dependent insulinotropic receptor	0
P23978	Sodium- and chloride-dependent GABA transporter 1	0
O15379	Histone deacetylase 3	0
Q05469	Hormone-sensitive lipase	0
Q9Y271	Cysteinyl leukotriene receptor 1	0
P35916	Vascular endothelial growth factor receptor 3	0
Q86V86	Serine/threonine-protein kinase pim-3	0
Q16602	Calcitonin gene-related peptide type 1 receptor	0
P08172	Muscarinic acetylcholine receptor M2	0
P08173	Muscarinic acetylcholine receptor M4	0
P22303	Acetylcholinesterase	0
P32245	Melanocortin receptor 4	0
P53779	Mitogen-activated protein kinase 10	0
P32247	Bombesin receptor subtype-3	0
P32246	C-C chemokine receptor type 1	0
P00491	Purine nucleoside phosphorylase	0
P43115	Prostaglandin E2 receptor EP3 subtype	0
P47820	Angiotensin-converting enzyme	0
P43116	Prostaglandin E2 receptor EP2 subtype	0
P35346	Somatostatin receptor type 5	0
P00797	Renin	0
P27487	Dipeptidyl peptidase 4	0
O14684	Prostaglandin E synthase	0
P35348	Alpha-1A adrenergic receptor	0
P78536	Disintegrin and metalloproteinase domain-containing protein 17	0
P16184	Dihydrofolate reductase	0

O15530	3-phosphoinositide-dependent protein kinase 1	0
P13516	Acyl-CoA desaturase 1	0
Q00975	Voltage-dependent N-type calcium channel subunit alpha-1B	0
P32745	Somatostatin receptor type 3	0
P35351	Type-2 angiotensin II receptor	0
P50172	Corticosteroid 11-beta-dehydrogenase isozyme 1	0
P33261	Cytochrome P450 2C19	0
P08254	Stromelysin-1	0
Q64663	P2X purinoceptor 7	0
P35462	D(3) dopamine receptor	0
P35463	Endothelin B receptor	0
P47898	5-hydroxytryptamine receptor 5A	0
Q9Y5X4	Photoreceptor-specific nuclear receptor	0
P04150	Glucocorticoid receptor	0
Q99720	Sigma non-opioid intracellular receptor 1	0
Q13627	Dual specificity tyrosine-phosphorylation-regulated kinase 1A	0
P34969	5-hydroxytryptamine receptor 7	0
Q01959	Sodium-dependent dopamine transporter	0
P20292	Arachidonate 5-lipoxygenase-activating protein	0
P08482	Muscarinic acetylcholine receptor M1	0
P30560	Vasopressin V1a receptor	0
P47871	Glucagon receptor	0
O96020	G1/S-specific cyclin-E2	0
P23975	Sodium-dependent noradrenaline transporter	0
P23977	Sodium-dependent dopamine transporter	0
O96017	Serine/threonine-protein kinase Chk2	0
P41231	P2Y purinoceptor 2	0
O96013	Serine/threonine-protein kinase PAK 4	0
P41235	Hepatocyte nuclear factor 4-alpha	0
P19156	Potassium-transporting ATPase alpha chain 1	0
P56524	Histone deacetylase 4	0
P18405	3-oxo-5-alpha-steroid 4-dehydrogenase 1	0
Q96RI1	Bile acid receptor	0
Q6V1X1	Dipeptidyl peptidase 8	0
O95136	Sphingosine 1-phosphate receptor 2	0
P55211	Caspase-9	0
P55210	Caspase-7	0
P55212	Caspase-6	0
O77636	Disintegrin and metalloproteinase domain-containing protein 17	0
P51681	C-C chemokine receptor type 5	0
P28845	Corticosteroid 11-beta-dehydrogenase isozyme 1	0
P51685	C-C chemokine receptor type 8	0
P31648	Sodium- and chloride-dependent GABA transporter 1	0
P08909	5-hydroxytryptamine receptor 2C	0
P08908	5-hydroxytryptamine receptor 1A	0
Q07343	cAMP-specific 3',5'-cyclic phosphodiesterase 4B	0
P31645	Sodium-dependent serotonin transporter	0
Q96RJ0	Trace amine-associated receptor 1	0
P31424	Metabotropic glutamate receptor 5	0
P31389	Histamine H1 receptor	0
P31388	5-hydroxytryptamine receptor 6	0

P18901	D(1A) dopamine receptor	0
P56481	Gastrin/cholecystokinin type B receptor	0
Q60492	Sigma non-opioid intracellular receptor 1	0
P15144	Aminopeptidase N	0
P05129	Protein kinase C gamma type	0
P09483	Neuronal acetylcholine receptor subunit alpha-4	0
P07948	Tyrosine-protein kinase Lyn	0
P07949	Proto-oncogene tyrosine-protein kinase receptor Ret	0
O95180	Voltage-dependent T-type calcium channel subunit alpha-1H	0
P16581	E-selectin	0
Q969F8	KiSS-1 receptor	0
O00748	Cocaine esterase	0
P14780	Matrix metalloproteinase-9	0
Q3KRE8	Tubulin beta-2B chain	0
Q16548	Bcl-2-related protein A1	0
P11387	DNA topoisomerase 1	0
P29466	Caspase-1	0
P61073	C-X-C chemokine receptor type 4	0
P28482	Mitogen-activated protein kinase 1	0
Q14432	cGMP-inhibited 3',5'-cyclic phosphodiesterase A	0
P06493	Cyclin-dependent kinase 1	0
P08238	Heat shock protein HSP 90-beta	0
Q95136	D(1A) dopamine receptor	0
P28335	5-hydroxytryptamine receptor 2C	0
Q9HAZ1	Dual specificity protein kinase CLK4	0
Q9UNQ0	ATP-binding cassette sub-family G member 2	0
P36888	Receptor-type tyrosine-protein kinase FLT3	0
P13945	Beta-3 adrenergic receptor	0
P00918	Carbonic anhydrase 2	0
O95822	Malonyl-CoA decarboxylase, mitochondrial	0
P20648	Potassium-transporting ATPase alpha chain 1	0
P07308	Acyl-CoA desaturase 1	0
O70536	Sterol O-acyltransferase 1	0
P48067	Sodium- and chloride-dependent glycine transporter 1	0
O00519	Fatty-acid amide hydrolase 1	0
P00915	Carbonic anhydrase 1	0
Q9QYJ6	cAMP and cAMP-inhibited cGMP 3',5'-cyclic phosphodiesterase 10A	0
P21731	Thromboxane A2 receptor	0
P35398	Nuclear receptor ROR-alpha	0
Q8TDU6	G-protein coupled bile acid receptor 1	0
P08514	Integrin alpha-IIb	0
P43166	Carbonic anhydrase 7	0
P10635	Cytochrome P450 2D6	0
P49146	Neuropeptide Y receptor type 2	0
P30530	Tyrosine-protein kinase receptor UFO	0
P46663	B1 bradykinin receptor	0
P32238	Cholecystokinin receptor type A	0
P32239	Gastrin/cholecystokinin type B receptor	0
P35236	Tyrosine-protein phosphatase non-receptor type 7	0
P30874	Somatostatin receptor type 2	0
P15538	Cytochrome P450 11B1, mitochondrial	0
P30872	Somatostatin receptor type 1	0

Q9HC97	G-protein coupled receptor 35	0
P14324	Farnesyl pyrophosphate synthase	0
P16257	Translocator protein	0
P42785	Lysosomal Pro-X carboxypeptidase	0
Q13547	Histone deacetylase 1	0
P06239	Tyrosine-protein kinase Lck	0
P50750	Cyclin-dependent kinase 9	0
P68400	Casein kinase II subunit alpha	0
P22086	Alpha-2C adrenergic receptor	0
P37136	Acetylcholinesterase	0
P31751	RAC-beta serine/threonine-protein kinase	0
P32305	5-hydroxytryptamine receptor 7	0
P33533	Delta-type opioid receptor	0
P32300	Delta-type opioid receptor	0
P33535	Mu-type opioid receptor	0
P39900	Macrophage metalloelastase	0
P24557	Thromboxane-A synthase	0
Q9GZT9	Egl nine homolog 1	0
P13726	Tissue factor	0
P35439	Glutamate receptor ionotropic, NMDA 1	0
P14061	Estradiol 17-beta-dehydrogenase 1	0
P50130	D(1A) dopamine receptor	0
Q8WW43	Gamma-secretase subunit APH-1B	0
P20309	Muscarinic acetylcholine receptor M3	0
Q8T6T2	Inosine-5'-monophosphate dehydrogenase	0
P07607	Thymidylate synthase	0
P42262	Glutamate receptor 2	0
P51661	Corticosteroid 11-beta-dehydrogenase isozyme 2	0
P18825	Alpha-2C adrenergic receptor	0
Q923Y8	Trace amine-associated receptor 1	0
P97266	Mu-type opioid receptor	0
P19020	D(3) dopamine receptor	0
P03956	Interstitial collagenase	0
P10415	Apoptosis regulator Bcl-2	0
P55263	Adenosine kinase	0
P07861	Neprilysin	0
O00311	Cell division cycle 7-related protein kinase	0
P31421	Metabotropic glutamate receptor 2	0
Q99685	Monoglyceride lipase	0
P02550	Tubulin alpha-1A chain	0
O60240	Perilipin-1	0
P11229	Muscarinic acetylcholine receptor M1	0
P12527	Arachidonate 5-lipoxygenase	0
Q05655	Protein kinase C delta type	0
P60953	Cell division control protein 42 homolog	0
P13612	Integrin alpha-4	0
O14842	Free fatty acid receptor 1	0
Q15661	Tryptase alpha/beta-1	0
P51812	Ribosomal protein S6 kinase alpha-3	0
O43353	Receptor-interacting serine/threonine-protein kinase 2	0
P21836	Acetylcholinesterase	0
O14920	Inhibitor of nuclear factor kappa-B kinase subunit beta	0
P49430	Thromboxane-A synthase	0
O75116	Rho-associated protein kinase 2	0

Q15858	Sodium channel protein type 9 subunit alpha	0
Q9NZ42	Gamma-secretase subunit PEN-2	0
P11086	Phenylethanolamine N-methyltransferase	0
P07943	Aldose reductase	0
P11712	Cytochrome P450 2C9	0
P47811	Mitogen-activated protein kinase 14	0
Q05769	Prostaglandin G/H synthase 2	0
P35372	Mu-type opioid receptor	0
Q8N1Q1	Carbonic anhydrase 13	0
Q86TI2	Dipeptidyl peptidase 9	0
P08246	Neutrophil elastase	0
Q9H3N8	Histamine H4 receptor	0
P17948	Vascular endothelial growth factor receptor 1	0
Q92769	Histone deacetylase 2	0
P55157	Microsomal triglyceride transfer protein large subunit	0
Q9NWZ3	Interleukin-1 receptor-associated kinase 4	0
P05121	Plasminogen activator inhibitor 1	0
Q96BI3	Gamma-secretase subunit APH-1A	0
Q92542	Nicastrin	0
P37231	Peroxisome proliferator-activated receptor gamma	0
P42338	Phosphatidylinositol 4,5-bisphosphate 3-kinase catalytic subunit beta isoform	0
P42336	Phosphatidylinositol 4,5-bisphosphate 3-kinase catalytic subunit alpha isoform	0
P45452	Collagenase 3	0
P22894	Neutrophil collagenase	0
P42330	Aldo-keto reductase family 1 member C3	0
P15917	Lethal factor	0
P30989	Neurotensin receptor type 1	0
Q08499	cAMP-specific 3',5'-cyclic phosphodiesterase 4D	0
P81908	Cholinesterase	0
Q14833	Metabotropic glutamate receptor 4	0
P00734	Prothrombin	0
P00735	Prothrombin	0
P23946	Chymase	0
O14746	Telomerase reverse transcriptase	0
P34975	Kappa-type opioid receptor	0
P34976	Type-1 angiotensin II receptor	0
O60755	Galanin receptor type 3	0
Q9Y233	cAMP and cAMP-inhibited cGMP 3',5'-cyclic phosphodiesterase 10A	0
P50406	5-hydroxytryptamine receptor 6	0
P43235	Cathepsin K	0
Q99808	Equilibrative nucleoside transporter 1	0
P43681	Neuronal acetylcholine receptor subunit alpha-4	0
P28190	Adenosine receptor A1	0
Q9UBN7	Histone deacetylase 6	0
P50613	Cyclin-dependent kinase 7	0
Q9UHL4	Dipeptidyl peptidase 2	0
P63000	Ras-related C3 botulinum toxin substrate 1	0
P48443	Retinoic acid receptor RXR-gamma	0
Q00987	E3 ubiquitin-protein ligase Mdm2	0
P46925	Plasmepsin-2	0

Q63470	Dual specificity tyrosine-phosphorylation-regulated kinase 1A	0
P05771	Protein kinase C beta type	0
P11309	Serine/threonine-protein kinase pim-1	0
Q9Y2D0	Carbonic anhydrase 5B, mitochondrial	0
Q13093	Platelet-activating factor acetylhydrolase	0
P06756	Integrin alpha-V	0
P31652	Sodium-dependent serotonin transporter	0
P20231	Tryptase beta-2	0
O14965	Aurora kinase A	0
P62943	Peptidyl-prolyl cis-trans isomerase FKBP1A	0
P62942	Peptidyl-prolyl cis-trans isomerase FKBP1A	0
P41597	C-C chemokine receptor type 2	0
Q99572	P2X purinoceptor 7	0
P16753	Capsid scaffolding protein	0
P09874	Poly [ADP-ribose] polymerase 1	0
P07384	Calpain-1 catalytic subunit	0
P14842	5-hydroxytryptamine receptor 2A	0
O00329	Phosphatidylinositol 4,5-bisphosphate 3-kinase catalytic subunit delta isoform	0
P22748	Carbonic anhydrase 4	0
P27815	cAMP-specific 3',5'-cyclic phosphodiesterase 4A	0
Q16539	Mitogen-activated protein kinase 14	0
Q28156	cGMP-specific 3',5'-cyclic phosphodiesterase	0
Q9JI35	Histamine H3 receptor	0
P30968	Gonadotropin-releasing hormone receptor	0
P30969	Gonadotropin-releasing hormone receptor	0
P11597	Cholesteryl ester transfer protein	0
P23280	Carbonic anhydrase 6	0
Q13224	Glutamate receptor ionotropic, NMDA 2B	0
Q07817	Bcl-2-like protein 1	0
P09237	Matrilysin	0
Q07869	Peroxisome proliferator-activated receptor alpha	0
P00519	Tyrosine-protein kinase ABL1	0
P05186	Alkaline phosphatase, tissue-nonspecific isozyme	0
P04818	Thymidylate synthase	0
P00517	cAMP-dependent protein kinase catalytic subunit alpha	0
Q9Y5N1	Histamine H3 receptor	0
O95977	Sphingosine 1-phosphate receptor 4	0
O75907	Diacylglycerol O-acyltransferase 1	0
Q29010	Endothelin-1 receptor	0
Q05941	Neuronal acetylcholine receptor subunit alpha-7	0
Q99835	Smoothened homolog	0
P49768	Presenilin-1	0
Q9NR96	Toll-like receptor 9	0
P13922	Bifunctional dihydrofolate reductase-thymidylate synthase	0
P09958	Furin	0
P15056	Serine/threonine-protein kinase B-raf	0
Q9UKP6	Urotensin-2 receptor	0
P06241	Tyrosine-protein kinase Fyn	0
P42574	Caspase-3	0
P21554	Cannabinoid receptor 1	0
P37059	Estradiol 17-beta-dehydrogenase 2	0

P37058	Testosterone 17-beta-dehydrogenase 3	0
