# Supplementary material for: Ginsenoside Rg1 as a Potential Regulator of Hematopoietic Stem/Progenitor Cells
Source: Stem Cells Int. 2021 Dec 31;2021:4633270. doi: 10.1155/2021/4633270 (PMC8741398; doi:10.1155/2021/4633270)
Supplement: Supplementary Materials — Supplementary Material 1: putative targets of Rg1 identified using TargetNet. Supplementary Material 2: putative targets of Rg1 identified using SwissTargetPrediction. Supplementary Material 3: genes involved in HSC proliferation. Supplementary Material 4: genes involved in HSC migration. Supplementary Material 5: genes involved in HSC differentiation. Supplementary Material 6: genes involved in HPC differentiation. Supplementary Material 7: databases used in this study. [file 4633270.f1.zip › Supplementary Material 2.pdf]

SwissTargetPrediction

Target	Common name	Uniprot ID	ChEMBL ID	Target Class	Probability*	Known actives (3D/2D)
Signal transducer and activator of transcription 3	STAT3	P40763	CHEMBL4026	Transcription factor	0.119923828	4 / 7
Interleukin-2	IL2	P60568	CHEMBL5880	Secreted protein	0.02205633	0 / 1
Platelet activating factor receptor	PTAFR	P25105	CHEMBL250	Family A G protein-coupled receptor	0.02205633	0 / 11
Vascular endothelial growth factor A	VEGFA	P15692	CHEMBL1783	Secreted protein	0.02205633	0 / 2
Acidic fibroblast growth factor	FGF1	P05230	CHEMBL2120	Secreted protein	0.02205633	0 / 5
Basic fibroblast growth factor	FGF2	P09038	CHEMBL3107	Secreted protein	0.02205633	0 / 2
Heparanase	HPSE	Q9Y251	CHEMBL3921	Enzyme	0.02205633	0 / 4
Sodium/potassium-transporting ATPase alpha-1 chain	ATP1A1	P05023	CHEMBL1807	Primary active transporter	0.02205633	0 / 1
Heat shock protein HSP 90-alpha	HSP90AA1	P07900	CHEMBL3880	Other cytosolic protein	0.02205633	5 / 2
Thymidylate synthase (by homology)	TYMS	P04818	CHEMBL1952	Transferase	0.02205633	90 / 0
Neurokinin 2 receptor	TACR2	P21452	CHEMBL2327	Family A G protein-coupled receptor	0.02205633	7 / 0
Integrin alpha-V/beta-3	ITGAV ITGB3	P06756 P05106	CHEMBL1907598	Membrane receptor	0.02205633	197 / 0
Integrin alpha-IIb/beta-3	ITGA2B ITGB3	P08514 P05106	CHEMBL2093869	Membrane receptor	0.02205633	229 / 0
Galectin-4	LGALS4	P56470	CHEMBL1671608	Other cytosolic protein	0.02205633	0 / 2
Galectin-3	LGALS3	P17931	CHEMBL4531	Other cytosolic protein	0.02205633	0 / 3
Galectin-8	LGALS8	O00214	CHEMBL5475	Other cytosolic protein	0.02205633	0 / 2
Serine/threonine-protein kinase AKT2	AKT2	P31751	CHEMBL2431	Kinase	0	2 / 0
Ribosomal protein S6 kinase alpha 1	RPS6KA1	Q15418	CHEMBL2553	Kinase	0	1 / 0
Rho-associated protein kinase 1	ROCK1	Q13464	CHEMBL3231	Kinase	0	1 / 0
Serine/threonine-protein kinase AKT	AKT1	P31749	CHEMBL4282	Kinase	0	2 / 0
Gamma-secretase	PSEN2 PSENEN NCSTN APH1A PSEN1 APH1B	P49810 Q9NZ42 Q92542 Q96BI3 P49768 Q8WW43	CHEMBL2094135	Protease	0	2 / 7
Receptor-type tyrosine-protein phosphatase alpha	PTPRA	P18433	CHEMBL3918	Phosphatase	0	7 / 0
Delta opioid receptor (by homology)	OPRD1	P41143	CHEMBL236	Family A G protein-coupled receptor	0	242 / 0
11-beta-hydroxysteroid dehydrogenase 2	HSD11B2	P80365	CHEMBL3746	Enzyme	0	0 / 1
11-beta-hydroxysteroid dehydrogenase 1	HSD11B1	P28845	CHEMBL4235	Enzyme	0	0 / 4
c-Jun N-terminal kinase 1	MAPK8	P45983	CHEMBL2276	Kinase	0	4 / 0
Renin	REN	P00797	CHEMBL286	Protease	0	33 / 0
c-Jun N-terminal kinase 2	MAPK9	P45984	CHEMBL4179	Kinase	0	3 / 0
Insulin-like growth factor I receptor	IGF1R	P08069	CHEMBL1957	Kinase	0	5 / 0
Epidermal growth factor receptor erbB1	EGFR	P00533	CHEMBL203	Kinase	0	10 / 0
Calpain 1	CAPN1	P07384	CHEMBL3891	Protease	0	3 / 0
Cystinyl aminopeptidase	LNPEP	Q9UIQ6	CHEMBL2693	Protease	0	35 / 0
Sphingosine 1-phosphate receptor Edg-1	S1PR1	P21453	CHEMBL4333	Family A G protein-coupled receptor	0	18 / 0
Peroxisome proliferator-activated receptor alpha	PPARA	Q07869	CHEMBL239	Nuclear receptor	0	8 / 0
Sodium/glucose cotransporter 2	SLC5A2	P31639	CHEMBL3884	Electrochemical transporter	0	21 / 0
Sodium/glucose cotransporter 1	SLC5A1	P13866	CHEMBL4979	Electrochemical transporter	0	16 / 0
Thrombin and coagulation factor X	F10	P00742	CHEMBL244	Protease	0	32 / 0
Serine/threonine-protein kinase mTOR	MTOR	P42345	CHEMBL2842	Kinase	0	22 / 0
Glucose-6-phosphate translocase	SLC37A4	O43826	CHEMBL3217398	Electrochemical transporter	0	1 / 0
Dipeptidyl peptidase IV	DPP4	P27487	CHEMBL284	Protease	0	21 / 0
Growth factor receptor-bound protein 2	GRB2	P62993	CHEMBL3663	Other cytosolic protein	0	27 / 0
Bcl-2-related protein A1 (by homology)	BCL2A1	Q16548	CHEMBL6044	Unclassified protein	0	2 / 0
Beta-secretase 1	BACE1	P56817	CHEMBL4822	Protease	0	47 / 0
Integrin alpha-5/beta-1	ITGB1 ITGA5	P05556 P08648	CHEMBL2095226	Membrane receptor	0	34 / 0
Integrin alpha-V/beta-5	ITGB5 ITGAV	P18084 P06756	CHEMBL2096675	Membrane receptor	0	53 / 0
Integrin alpha-V/beta-6	ITGAV ITGB6	P06756 P18564	CHEMBL2111416	Membrane receptor	0	22 / 0
Histone deacetylase 3	HDAC3	O15379	CHEMBL1829	Eraser	0	8 / 0
Histone deacetylase 6	HDAC6	Q9UBN7	CHEMBL1865	Eraser	0	19 / 0
Epoxide hydratase	EPHX2	P34913	CHEMBL2409	Protease	0	1 / 0
Histone deacetylase 8	HDAC8	Q9BY41	CHEMBL3192	Eraser	0	8 / 0
Histone deacetylase 1	HDAC1	Q13547	CHEMBL325	Eraser	0	28 / 0
Inhibitor of apoptosis protein 3	XIAP	P98170	CHEMBL4198	Other cytosolic protein	0	28 / 0
Dual specificity mitogen-activated protein kinase kinase 1	MAP2K1	Q02750	CHEMBL3587	Kinase	0	4 / 0
Adrenergic receptor beta	ADRB2	P07550	CHEMBL210	Family A G protein-coupled receptor	0	10 / 0
Matrix metalloproteinase 13	MMP13	P45452	CHEMBL280	Protease	0	14 / 0
Matrix metalloproteinase 2	MMP2	P08253	CHEMBL333	Protease	0	16 / 0
Leukotriene B4 receptor 1	LTB4R	Q15722	CHEMBL3911	Family A G protein-coupled receptor	0	26 / 0
Nuclear receptor ROR-gamma	RORC	P51449	CHEMBL1741186	Nuclear receptor	0	1 / 2
Cyclophilin A	PPIA	P62937	CHEMBL1949	Isomerase	0	6 / 0
Matrix metalloproteinase 1	MMP1	P03956	CHEMBL332	Protease	0	5 / 0
Matrix metalloproteinase 12	MMP12	P39900	CHEMBL4393	Protease	0	5 / 0
Protein-tyrosine phosphatase 1B	PTPN1	P18031	CHEMBL335	Phosphatase	0	60 / 25
Plasminogen	PLG	P00747	CHEMBL1801	Protease	0	4 / 0
Trypsin I	PRSS1	P07477	CHEMBL209	Protease	0	12 / 0
Adenosine kinase	ADK	P55263	CHEMBL3589	Enzyme	0	5 / 0
C3a anaphylatoxin chemotactic receptor	C3AR1	Q16581	CHEMBL4761	Family A G protein-coupled receptor	0	4 / 0
PI3-kinase p110-alpha subunit	PIK3CA	P42336	CHEMBL4005	Enzyme	0	14 / 0
Macrophage colony stimulating factor receptor	CSF1R	P07333	CHEMBL1844	Kinase	0	8 / 0
Integrin alpha-4	ITGA4	P13612	CHEMBL278	Membrane receptor	0	10 / 0
Nerve growth factor receptor Trk-A	NTRK1	P04629	CHEMBL2815	Kinase	0	2 / 0
Neurotrophic tyrosine kinase receptor type 2	NTRK2	Q16620	CHEMBL4898	Kinase	0	1 / 0
Tyrosine-protein kinase SYK	SYK	P43405	CHEMBL2599	Kinase	0	11 / 0
G protein-coupled receptor 44	PTGDR2	Q9Y5Y4	CHEMBL5071	Family A G protein-coupled receptor	0	2 / 0
Adenosine A1 receptor (by homology)	ADORA1	P30542	CHEMBL226	Family A G protein-coupled receptor	0	35 / 0
Rho-associated protein kinase 2	ROCK2	O75116	CHEMBL2973	Kinase	0	5 / 0
N-lysine methyltransferase SETD8	KMT5A	Q9NQR1	CHEMBL1795176	Writer	0	1 / 0
Leukocyte adhesion glycoprotein LFA-1 alpha	ITGAL	P20701	CHEMBL1803	Adhesion	0	3 / 0
Caspase-3	CASP3	P42574	CHEMBL2334	Protease	0	30 / 0
Peroxisome proliferator-activated receptor gamma	PPARG	P37231	CHEMBL235	Nuclear receptor	0	19 / 0
Apoptosis regulator Bcl-X	BCL2L1	Q07817	CHEMBL4625	Other ion channel	0	1 / 1
Dihydroorotate dehydrogenase	DHODH	Q02127	CHEMBL1966	Oxidoreductase	0	4 / 0
Sphingosine 1-phosphate receptor Edg-3	S1PR3	Q99500	CHEMBL3892	Family A G protein-coupled receptor	0	11 / 0
Endothelin-converting enzyme 1	ECE1	P42892	CHEMBL4791	Protease	0	3 / 0
Aminopeptidase N	ANPEP	P15144	CHEMBL1907	Protease	0	19 / 0
Beta amyloid A4 protein	APP	P05067	CHEMBL2487	Membrane receptor	0	5 / 0
Receptor-type tyrosine-protein phosphatase F (LAR)	PTPRF	P10586	CHEMBL3521	Membrane receptor	0	1 / 0
Integrin alpha-4/beta-1	ITGB1 ITGA4	P05556 P13612	CHEMBL1907599	Membrane receptor	0	33 / 0
Type-1 angiotensin II receptor (by homology)	AGTR1	P30556	CHEMBL227	Family A G protein-coupled receptor	0	7 / 0
Leukocyte adhesion molecule-1	SELL	P14151	CHEMBL3161	Adhesion	0	3 / 0
Hydroxycarboxylic acid receptor 2	HCAR2	Q8TDS4	CHEMBL3785	Family A G protein-coupled receptor	0	35 / 0
P-selectin	SELP	P16109	CHEMBL5378	Adhesion	0	3 / 0
Serotonin 2b (5-HT2b) receptor	HTR2B	P41595	CHEMBL1833	Family A G protein-coupled receptor	0	0 / 1
Alpha-2a adrenergic receptor	ADRA2A	P08913	CHEMBL1867	Family A G protein-coupled receptor	0	0 / 1
Adrenergic receptor alpha-2	ADRA2C	P18825	CHEMBL1916	Family A G protein-coupled receptor	0	0 / 1
Alpha-2b adrenergic receptor	ADRA2B	P18089	CHEMBL1942	Family A G protein-coupled receptor	0	0 / 1
Dopamine D1 receptor	DRD1	P21728	CHEMBL2056	Family A G protein-coupled receptor	0	0 / 1
Alpha-1d adrenergic receptor	ADRA1D	P25100	CHEMBL223	Family A G protein-coupled receptor	0	0 / 1
Cytochrome P450 2D6	CYP2D6	P10635	CHEMBL289	Cytochrome P450	0	0 / 1
Serotonin 6 (5-HT6) receptor	HTR6	P50406	CHEMBL3371	Family A G protein-coupled receptor	0	0 / 1
Serotonin 1b (5-HT1b) receptor (by homology)	HTR1B	P28222	CHEMBL1898	Family A G protein-coupled receptor	0	0 / 1
