# Supplementary material for: Ginsenoside Rg1 as a Potential Regulator of Hematopoietic Stem/Progenitor Cells
Source: Stem Cells Int. 2021 Dec 31;2021:4633270. doi: 10.1155/2021/4633270 (PMC8741398; doi:10.1155/2021/4633270)
Supplement: Supplementary Materials — Supplementary Material 1: putative targets of Rg1 identified using TargetNet. Supplementary Material 2: putative targets of Rg1 identified using SwissTargetPrediction. Supplementary Material 3: genes involved in HSC proliferation. Supplementary Material 4: genes involved in HSC migration. Supplementary Material 5: genes involved in HSC differentiation. Supplementary Material 6: genes involved in HPC differentiation. Supplementary Material 7: databases used in this study. [file 4633270.f1.zip › Supplementary Material 3.pdf]

GO Term Name	GO Term ID	Gene Symbol	Gene ID
hematopoietic stem cell proliferation	GO:0071425	ARIH2	10425
hematopoietic stem cell proliferation	GO:0071425	BABAM1	29086
hematopoietic stem cell proliferation	GO:0071425	CD34	947
hematopoietic stem cell proliferation	GO:0071425	CSF3A	100270759
hematopoietic stem cell proliferation	GO:0071425	CSF3B	100190920
hematopoietic stem cell proliferation	GO:0071425	CTC1	80169
hematopoietic stem cell proliferation	GO:0071425	CYP27B1	1594
hematopoietic stem cell proliferation	GO:0071425	CYP2R1	120227
hematopoietic stem cell proliferation	GO:0071425	DAB2	1601
hematopoietic stem cell proliferation	GO:0071425	ETV6	2120
hematopoietic stem cell proliferation	GO:0071425	FUBP1	8880
hematopoietic stem cell proliferation	GO:0071425	GBA	2629
hematopoietic stem cell proliferation	GO:0071425	ISM1	140862
hematopoietic stem cell proliferation	GO:0071425	MECOM	2122
hematopoietic stem cell proliferation	GO:0071425	METTL3	56339
hematopoietic stem cell proliferation	GO:0071425	MIR143	406935
hematopoietic stem cell proliferation	GO:0071425	MIR145	406937
hematopoietic stem cell proliferation	GO:0071425	MIR145A	387163
hematopoietic stem cell proliferation	GO:0071425	MIR145B	102465187
hematopoietic stem cell proliferation	GO:0071425	NKAP	79576
hematopoietic stem cell proliferation	GO:0071425	PRG4	10216
hematopoietic stem cell proliferation	GO:0071425	RAD51	5888
hematopoietic stem cell proliferation	GO:0071425	RUNX1	861
hematopoietic stem cell proliferation	GO:0071425	SART3	9733
hematopoietic stem cell proliferation	GO:0071425	SFRP2	6423
hematopoietic stem cell proliferation	GO:0071425	SHB	6461
hematopoietic stem cell proliferation	GO:0071425	TFEC	22797
hematopoietic stem cell proliferation	GO:0071425	VDRA	30076
hematopoietic stem cell proliferation	GO:0071425	WNT1	7471
hematopoietic stem cell proliferation	GO:0071425	WNT10B	7480
hematopoietic stem cell proliferation	GO:0071425	WNT2B	7482
hematopoietic stem cell proliferation	GO:0071425	WNT5A	7474
hematopoietic stem cell proliferation	GO:0071425	YJEFN3	374887
hematopoietic stem cell proliferation	GO:0071425	YTHDF2	51441
negative regulation of hematopoietic stem cell proliferation	GO:1902034	MIR221	407006
negative regulation of hematopoietic stem cell proliferation	GO:1902034	MIR222	407007
positive regulation of hematopoietic stem cell proliferation	GO:1902035	ATXN1L	342371
positive regulation of hematopoietic stem cell proliferation	GO:1902035	CXCL1	2919
positive regulation of hematopoietic stem cell proliferation	GO:1902035	KAT7	11143
positive regulation of hematopoietic stem cell proliferation	GO:1902035	KITL	17311
positive regulation of hematopoietic stem cell proliferation	GO:1902035	KITLG	4254
positive regulation of hematopoietic stem cell proliferation	GO:1902035	N4BP2L2	10443
positive regulation of hematopoietic stem cell proliferation	GO:1902035	PDCD2	5134
positive regulation of hematopoietic stem cell proliferation	GO:1902035	THPO	7066
regulation of hematopoietic stem cell proliferation	GO:1902033	ACE	1636
regulation of hematopoietic stem cell proliferation	GO:1902033	CCL25B	795788
regulation of hematopoietic stem cell proliferation	GO:1902033	EIF2AK2	5610
regulation of hematopoietic stem cell proliferation	GO:1902033	KLF6A	280650
regulation of hematopoietic stem cell proliferation	GO:1902033	PIM1	5292
