# Supplementary material for: Ginsenoside Rg1 as a Potential Regulator of Hematopoietic Stem/Progenitor Cells
Source: Stem Cells Int. 2021 Dec 31;2021:4633270. doi: 10.1155/2021/4633270 (PMC8741398; doi:10.1155/2021/4633270)
Supplement: Supplementary Materials — Supplementary Material 1: putative targets of Rg1 identified using TargetNet. Supplementary Material 2: putative targets of Rg1 identified using SwissTargetPrediction. Supplementary Material 3: genes involved in HSC proliferation. Supplementary Material 4: genes involved in HSC migration. Supplementary Material 5: genes involved in HSC differentiation. Supplementary Material 6: genes involved in HPC differentiation. Supplementary Material 7: databases used in this study. [file 4633270.f1.zip › Supplementary Material 4.pdf]

GO Term Name	GO Term ID	Gene Symbol	Gene ID
hematopoietic stem cell migration	GO:0035701	BCL11B	64919
hematopoietic stem cell migration	GO:0035701	BMI1A	321505
hematopoietic stem cell migration	GO:0035701	CEBPA	1050
hematopoietic stem cell migration	GO:0035701	CNR2	1269
hematopoietic stem cell migration	GO:0035701	CXCL12A	352944
hematopoietic stem cell migration	GO:0035701	CXCR4	7852
hematopoietic stem cell migration	GO:0035701	GPLD1	2822
hematopoietic stem cell migration	GO:0035701	KIT	3815
hematopoietic stem cell migration	GO:0035701	MMP2	4313
hematopoietic stem cell migration	GO:0035701	MMP9	4318
hematopoietic stem cell migration	GO:0035701	MTCH2	23788
hematopoietic stem cell migration	GO:0035701	MYB	4602
hematopoietic stem cell migration	GO:0035701	SELP	6403
hematopoietic stem cell migration	GO:0035701	SELPLG	6404
hematopoietic stem cell migration	GO:0035701	SOX32	116990
hematopoietic stem cell migration to bone marrow	GO:0097241	CNR2	1269
hematopoietic stem cell migration to bone marrow	GO:0097241	EXT1	2131
hematopoietic stem cell migration to bone marrow	GO:0097241	GAS6	2621
hematopoietic stem cell migration to bone marrow	GO:0097241	GPLD1	2822
hematopoietic stem cell migration to bone marrow	GO:0097241	JAM2	58494
hematopoietic stem cell migration to bone marrow	GO:0097241	JAM3	83700
hematopoietic stem cell migration to bone marrow	GO:0097241	SELP	6403
hematopoietic stem cell migration to bone marrow	GO:0097241	SELPLG	6404
negative regulation of hematopoietic stem cell migration	GO:2000472	CXCL12A	352944
positive regulation of hematopoietic stem cell migration	GO:2000473	CCR2	729230
positive regulation of hematopoietic stem cell migration	GO:2000473	PTPRC	5788
regulation of hematopoietic stem cell migration	GO:2000471	CCL25B	795788
regulation of hematopoietic stem cell migration	GO:2000471	KLF6A	280650
