# Supplementary material for: Ginsenoside Rg1 as a Potential Regulator of Hematopoietic Stem/Progenitor Cells
Source: Stem Cells Int. 2021 Dec 31;2021:4633270. doi: 10.1155/2021/4633270 (PMC8741398; doi:10.1155/2021/4633270)
Supplement: Supplementary Materials — Supplementary Material 1: putative targets of Rg1 identified using TargetNet. Supplementary Material 2: putative targets of Rg1 identified using SwissTargetPrediction. Supplementary Material 3: genes involved in HSC proliferation. Supplementary Material 4: genes involved in HSC migration. Supplementary Material 5: genes involved in HSC differentiation. Supplementary Material 6: genes involved in HPC differentiation. Supplementary Material 7: databases used in this study. [file 4633270.f1.zip › Supplementary Material 5.pdf]

GO Term Name	GO Term ID	Gene Symbol	Gene ID
hematopoietic stem cell differentiation	GO:0060218	ACE	1636
hematopoietic stem cell differentiation	GO:0060218	ADTRP1	550414
hematopoietic stem cell differentiation	GO:0060218	AK2	204
hematopoietic stem cell differentiation	GO:0060218	AP2A1	160
hematopoietic stem cell differentiation	GO:0060218	BATF	10538
hematopoietic stem cell differentiation	GO:0060218	BIF1.1	103908654
hematopoietic stem cell differentiation	GO:0060218	CDK6	1021
hematopoietic stem cell differentiation	GO:0060218	CHD2	1106
hematopoietic stem cell differentiation	GO:0060218	CNR2	1269
hematopoietic stem cell differentiation	GO:0060218	CSF3A	100270759
hematopoietic stem cell differentiation	GO:0060218	CSF3B	100190920
hematopoietic stem cell differentiation	GO:0060218	CXCL8A	100002946
hematopoietic stem cell differentiation	GO:0060218	DLC	30120
hematopoietic stem cell differentiation	GO:0060218	DLD	1738
hematopoietic stem cell differentiation	GO:0060218	EPAS1A	566886
hematopoietic stem cell differentiation	GO:0060218	EPAS1B	555192
hematopoietic stem cell differentiation	GO:0060218	ERCC2	2068
hematopoietic stem cell differentiation	GO:0060218	EXT1	2131
hematopoietic stem cell differentiation	GO:0060218	FEV	54738
hematopoietic stem cell differentiation	GO:0060218	FOSAB	394198
hematopoietic stem cell differentiation	GO:0060218	GATA2B	436962
hematopoietic stem cell differentiation	GO:0060218	HAL	3034
hematopoietic stem cell differentiation	GO:0060218	HDAC1	3065
hematopoietic stem cell differentiation	GO:0060218	HIF1AA	797150
hematopoietic stem cell differentiation	GO:0060218	HIF1AB	393202
hematopoietic stem cell differentiation	GO:0060218	HOXB4	3214
hematopoietic stem cell differentiation	GO:0060218	IL6	3569
hematopoietic stem cell differentiation	GO:0060218	JAG1A	140421
hematopoietic stem cell differentiation	GO:0060218	LMBR1L	55716
hematopoietic stem cell differentiation	GO:0060218	LRSAM1	90678
hematopoietic stem cell differentiation	GO:0060218	MEOX1	4222
hematopoietic stem cell differentiation	GO:0060218	METAP2B	323452
hematopoietic stem cell differentiation	GO:0060218	METTL22	79091
hematopoietic stem cell differentiation	GO:0060218	MIB1	57534
hematopoietic stem cell differentiation	GO:0060218	MIR125B1	406911
hematopoietic stem cell differentiation	GO:0060218	MIR125B-1	387236
hematopoietic stem cell differentiation	GO:0060218	MIR125B2	406912
hematopoietic stem cell differentiation	GO:0060218	MIR125B-2	723952
hematopoietic stem cell differentiation	GO:0060218	MIR126A	387145
hematopoietic stem cell differentiation	GO:0060218	MIR130A	406919
hematopoietic stem cell differentiation	GO:0060218	MIR142A	100033664
hematopoietic stem cell differentiation	GO:0060218	MIR155	406947
hematopoietic stem cell differentiation	GO:0060218	MIR181C	406957
hematopoietic stem cell differentiation	GO:0060218	MIR193B	574455
hematopoietic stem cell differentiation	GO:0060218	MIR542	664617
hematopoietic stem cell differentiation	GO:0060218	MIR99A	407055
hematopoietic stem cell differentiation	GO:0060218	MIRLET7E	406887
hematopoietic stem cell differentiation	GO:0060218	MLLT3	4300
hematopoietic stem cell differentiation	GO:0060218	MYCA	30686
hematopoietic stem cell differentiation	GO:0060218	NCOR2	9612
hematopoietic stem cell differentiation	GO:0060218	NOP14	8602
hematopoietic stem cell differentiation	GO:0060218	NOS1	4842
hematopoietic stem cell differentiation	GO:0060218	NOTCH1A	30718
hematopoietic stem cell differentiation	GO:0060218	NOTCH1B	794892
hematopoietic stem cell differentiation	GO:0060218	NPAS4L	108449885
hematopoietic stem cell differentiation	GO:0060218	PDCD2	5134
hematopoietic stem cell differentiation	GO:0060218	PDGFRB	5159
hematopoietic stem cell differentiation	GO:0060218	PLCG1	5335
hematopoietic stem cell differentiation	GO:0060218	PRDM16	63976
hematopoietic stem cell differentiation	GO:0060218	RPS29	6235
hematopoietic stem cell differentiation	GO:0060218	RSPO1	284654
hematopoietic stem cell differentiation	GO:0060218	SF3A3	10946

hematopoietic stem cell differentiation	GO:0060218	SF3B1	23451
hematopoietic stem cell differentiation	GO:0060218	SFRP1	6422
hematopoietic stem cell differentiation	GO:0060218	SNRNP70	6625
hematopoietic stem cell differentiation	GO:0060218	SP7	121340
hematopoietic stem cell differentiation	GO:0060218	SRF	6722
hematopoietic stem cell differentiation	GO:0060218	TAL1	6886
hematopoietic stem cell differentiation	GO:0060218	TBX16	30264
hematopoietic stem cell differentiation	GO:0060218	TERC	7012
hematopoietic stem cell differentiation	GO:0060218	TGFB1A	359834
hematopoietic stem cell differentiation	GO:0060218	TGFB1B	563884
hematopoietic stem cell differentiation	GO:0060218	TGFB3	7043
hematopoietic stem cell differentiation	GO:0060218	TGFBR2B	30739
hematopoietic stem cell differentiation	GO:0060218	TNNT2A	58071
hematopoietic stem cell differentiation	GO:0060218	TP53	7157
hematopoietic stem cell differentiation	GO:0060218	TRP53	22059
hematopoietic stem cell differentiation	GO:0060218	UFL1	23376
hematopoietic stem cell differentiation	GO:0060218	VHL	7428
hematopoietic stem cell differentiation	GO:0060218	WDR43	23160
hematopoietic stem cell differentiation	GO:0060218	WNT16	51384
hematopoietic stem cell differentiation	GO:0060218	WNT9A	7483
hematopoietic stem cell differentiation	GO:0060218	XRCC5	7520
negative regulation of hematopoietic stem cell differentiation	GO:1902037	ARF79F	40506
negative regulation of hematopoietic stem cell differentiation	GO:1902037	ASRIJ	37637
negative regulation of hematopoietic stem cell differentiation	GO:1902037	C1GALTA	34215
negative regulation of hematopoietic stem cell differentiation	GO:1902037	F2R	2149
negative regulation of hematopoietic stem cell differentiation	GO:1902037	HSPA9	3313
negative regulation of hematopoietic stem cell differentiation	GO:1902037	IRF7	3665
negative regulation of hematopoietic stem cell differentiation	GO:1902037	N4BP2L2	10443
negative regulation of hematopoietic stem cell differentiation	GO:1902037	NFE2L2	4780
negative regulation of hematopoietic stem cell differentiation	GO:1902037	OCIAD1	54940
negative regulation of hematopoietic stem cell differentiation	GO:1902037	OCIAD2	132299
positive regulation of hematopoietic stem cell differentiation	GO:1902038	BLOC1S2	282991
positive regulation of hematopoietic stem cell differentiation	GO:1902038	DDX46	9879
positive regulation of hematopoietic stem cell differentiation	GO:1902038	FOXC1	2296
positive regulation of hematopoietic stem cell differentiation	GO:1902038	NELFB	25920
positive regulation of hematopoietic stem cell differentiation	GO:1902038	NELFE	7936
positive regulation of hematopoietic stem cell differentiation	GO:1902038	SUPT5H	6829
regulation of hematopoietic stem cell differentiation	GO:1902036	ABL1	25
regulation of hematopoietic stem cell differentiation	GO:1902036	ADORA2B	136
regulation of hematopoietic stem cell differentiation	GO:1902036	AP2A2	161
regulation of hematopoietic stem cell differentiation	GO:1902036	CBFB	865
regulation of hematopoietic stem cell differentiation	GO:1902036	CDCA7A	550236
regulation of hematopoietic stem cell differentiation	GO:1902036	CDK6	1021
regulation of hematopoietic stem cell differentiation	GO:1902036	CRHB	492507
regulation of hematopoietic stem cell differentiation	GO:1902036	DLD	1738
regulation of hematopoietic stem cell differentiation	GO:1902036	DNMT3BB.1	317744
regulation of hematopoietic stem cell differentiation	GO:1902036	EIF2AK2	5610
regulation of hematopoietic stem cell differentiation	GO:1902036	F11R.1	323696
regulation of hematopoietic stem cell differentiation	GO:1902036	FGF10A	359830
regulation of hematopoietic stem cell differentiation	GO:1902036	FGFR2	2263
regulation of hematopoietic stem cell differentiation	GO:1902036	FGFR3	2261
regulation of hematopoietic stem cell differentiation	GO:1902036	FGFR4	2264
regulation of hematopoietic stem cell differentiation	GO:1902036	GATA1	2623
regulation of hematopoietic stem cell differentiation	GO:1902036	GATA2	2624
regulation of hematopoietic stem cell differentiation	GO:1902036	GATA3	2625
regulation of hematopoietic stem cell differentiation	GO:1902036	ITCH	83737
regulation of hematopoietic stem cell differentiation	GO:1902036	KMT2A	4297
regulation of hematopoietic stem cell differentiation	GO:1902036	LDB1	8861
regulation of hematopoietic stem cell differentiation	GO:1902036	LMO1	4004
regulation of hematopoietic stem cell differentiation	GO:1902036	LMO2	4005
regulation of hematopoietic stem cell differentiation	GO:1902036	METTL3	56339
regulation of hematopoietic stem cell differentiation	GO:1902036	MYB	4602
regulation of hematopoietic stem cell differentiation	GO:1902036	NR3C1	2908

regulation of hematopoietic stem cell differentiation	GO:1902036	OSM	5008
regulation of hematopoietic stem cell differentiation	GO:1902036	POMCA	353221
regulation of hematopoietic stem cell differentiation	GO:1902036	POMCB	100034412
regulation of hematopoietic stem cell differentiation	GO:1902036	PRKDC	5591
regulation of hematopoietic stem cell differentiation	GO:1902036	PSMA1	5682
regulation of hematopoietic stem cell differentiation	GO:1902036	PSMA2	5683
regulation of hematopoietic stem cell differentiation	GO:1902036	PSMA3	5684
regulation of hematopoietic stem cell differentiation	GO:1902036	PSMA4	5685
regulation of hematopoietic stem cell differentiation	GO:1902036	PSMA5	5686
regulation of hematopoietic stem cell differentiation	GO:1902036	PSMA6	5687
regulation of hematopoietic stem cell differentiation	GO:1902036	PSMA7	5688
regulation of hematopoietic stem cell differentiation	GO:1902036	PSMA8	143471
regulation of hematopoietic stem cell differentiation	GO:1902036	PSMB1	5689
regulation of hematopoietic stem cell differentiation	GO:1902036	PSMB10	5699
regulation of hematopoietic stem cell differentiation	GO:1902036	PSMB11	122706
regulation of hematopoietic stem cell differentiation	GO:1902036	PSMB2	5690
regulation of hematopoietic stem cell differentiation	GO:1902036	PSMB3	5691
regulation of hematopoietic stem cell differentiation	GO:1902036	PSMB4	5692
regulation of hematopoietic stem cell differentiation	GO:1902036	PSMB5	5693
regulation of hematopoietic stem cell differentiation	GO:1902036	PSMB6	5694
regulation of hematopoietic stem cell differentiation	GO:1902036	PSMB7	5695
regulation of hematopoietic stem cell differentiation	GO:1902036	PSMB8	5696
regulation of hematopoietic stem cell differentiation	GO:1902036	PSMB9	5698
regulation of hematopoietic stem cell differentiation	GO:1902036	PSMC1	5700
regulation of hematopoietic stem cell differentiation	GO:1902036	PSMC2	5701
regulation of hematopoietic stem cell differentiation	GO:1902036	PSMC3	5702
regulation of hematopoietic stem cell differentiation	GO:1902036	PSMC4	5704
regulation of hematopoietic stem cell differentiation	GO:1902036	PSMC5	5705
regulation of hematopoietic stem cell differentiation	GO:1902036	PSMC6	5706
regulation of hematopoietic stem cell differentiation	GO:1902036	PSMD1	5707
regulation of hematopoietic stem cell differentiation	GO:1902036	PSMD10	5716
regulation of hematopoietic stem cell differentiation	GO:1902036	PSMD11	5717
regulation of hematopoietic stem cell differentiation	GO:1902036	PSMD12	5718
regulation of hematopoietic stem cell differentiation	GO:1902036	PSMD13	5719
regulation of hematopoietic stem cell differentiation	GO:1902036	PSMD14	10213
regulation of hematopoietic stem cell differentiation	GO:1902036	PSMD2	5708
regulation of hematopoietic stem cell differentiation	GO:1902036	PSMD3	5709
regulation of hematopoietic stem cell differentiation	GO:1902036	PSMD4	5710
regulation of hematopoietic stem cell differentiation	GO:1902036	PSMD5	5711
regulation of hematopoietic stem cell differentiation	GO:1902036	PSMD6	9861
regulation of hematopoietic stem cell differentiation	GO:1902036	PSMD7	5713
regulation of hematopoietic stem cell differentiation	GO:1902036	PSMD8	5714
regulation of hematopoietic stem cell differentiation	GO:1902036	PSMD9	5715
regulation of hematopoietic stem cell differentiation	GO:1902036	PSME1	5720
regulation of hematopoietic stem cell differentiation	GO:1902036	PSME2	5721
regulation of hematopoietic stem cell differentiation	GO:1902036	PSME3	10197
regulation of hematopoietic stem cell differentiation	GO:1902036	PSME4	23198
regulation of hematopoietic stem cell differentiation	GO:1902036	PSMF1	9491
regulation of hematopoietic stem cell differentiation	GO:1902036	PUS7	54517
regulation of hematopoietic stem cell differentiation	GO:1902036	RUNX1	861
regulation of hematopoietic stem cell differentiation	GO:1902036	SETD1A	9739
regulation of hematopoietic stem cell differentiation	GO:1902036	TAL1	6886
regulation of hematopoietic stem cell differentiation	GO:1902036	TCF12	6938
regulation of hematopoietic stem cell differentiation	GO:1902036	TCF3	6929
regulation of hematopoietic stem cell differentiation	GO:1902036	TP73	7161
regulation of hematopoietic stem cell differentiation	GO:1902036	TPH1A	352943
regulation of hematopoietic stem cell differentiation	GO:1902036	TPH1B	415103
regulation of hematopoietic stem cell differentiation	GO:1902036	TPH2	121278
regulation of hematopoietic stem cell differentiation	GO:1902036	URB2	9816
regulation of hematopoietic stem cell differentiation	GO:1902036	WNT16	51384
regulation of hematopoietic stem cell differentiation	GO:1902036	YAP1	10413
regulation of hematopoietic stem cell differentiation	GO:1902036	YTHDF2	51441
