# Supplementary material for: Ginsenoside Rg1 as a Potential Regulator of Hematopoietic Stem/Progenitor Cells
Source: Stem Cells Int. 2021 Dec 31;2021:4633270. doi: 10.1155/2021/4633270 (PMC8741398; doi:10.1155/2021/4633270)
Supplement: Supplementary Materials — Supplementary Material 1: putative targets of Rg1 identified using TargetNet. Supplementary Material 2: putative targets of Rg1 identified using SwissTargetPrediction. Supplementary Material 3: genes involved in HSC proliferation. Supplementary Material 4: genes involved in HSC migration. Supplementary Material 5: genes involved in HSC differentiation. Supplementary Material 6: genes involved in HPC differentiation. Supplementary Material 7: databases used in this study. [file 4633270.f1.zip › Supplementary Material 6.pdf]

GO Term Name	GO Term ID	Gene Symbol	Gene ID
hematopoietic progenitor cell differentiation	GO:0002244	ABCA15	320631
hematopoietic progenitor cell differentiation	GO:0002244	ACP6	51205
hematopoietic progenitor cell differentiation	GO:0002244	ADAR	103
hematopoietic progenitor cell differentiation	GO:0002244	AGPAT5	55326
hematopoietic progenitor cell differentiation	GO:0002244	AK2	204
hematopoietic progenitor cell differentiation	GO:0002244	ANGPTL1A	544656
hematopoietic progenitor cell differentiation	GO:0002244	ANGPTL2B	503514
hematopoietic progenitor cell differentiation	GO:0002244	ANLN	54443
hematopoietic progenitor cell differentiation	GO:0002244	AP3B1	8546
hematopoietic progenitor cell differentiation	GO:0002244	APOBEC3	80287
hematopoietic progenitor cell differentiation	GO:0002244	ARHGEF7	8874
hematopoietic progenitor cell differentiation	GO:0002244	ARL11	115761
hematopoietic progenitor cell differentiation	GO:0002244	ARMC6	93436
hematopoietic progenitor cell differentiation	GO:0002244	ASH2L	9070
hematopoietic progenitor cell differentiation	GO:0002244	BMP4	652
hematopoietic progenitor cell differentiation	GO:0002244	BRD8B	337414
hematopoietic progenitor cell differentiation	GO:0002244	BVES	11149
hematopoietic progenitor cell differentiation	GO:0002244	C12ORF29	91298
hematopoietic progenitor cell differentiation	GO:0002244	C5H12ORF29	100933671
hematopoietic progenitor cell differentiation	GO:0002244	CBX6B	556231
hematopoietic progenitor cell differentiation	GO:0002244	CBX8B	799361
hematopoietic progenitor cell differentiation	GO:0002244	CDK6	1021
hematopoietic progenitor cell differentiation	GO:0002244	CEBPD	1052
hematopoietic progenitor cell differentiation	GO:0002244	CECR2	27443
hematopoietic progenitor cell differentiation	GO:0002244	CHD7	55636
hematopoietic progenitor cell differentiation	GO:0002244	CIAO3	64428
hematopoietic progenitor cell differentiation	GO:0002244	CITED2	10370
hematopoietic progenitor cell differentiation	GO:0002244	COL24A1	255631
hematopoietic progenitor cell differentiation	GO:0002244	CREBBPA	566841
hematopoietic progenitor cell differentiation	GO:0002244	CXCL8A	100002946
hematopoietic progenitor cell differentiation	GO:0002244	CXXC1A	393571
hematopoietic progenitor cell differentiation	GO:0002244	CYP2C66	69888
hematopoietic progenitor cell differentiation	GO:0002244	DACT2	168002
hematopoietic progenitor cell differentiation	GO:0002244	DHTKD1	55526
hematopoietic progenitor cell differentiation	GO:0002244	DLC	30120
hematopoietic progenitor cell differentiation	GO:0002244	DLD	1738
hematopoietic progenitor cell differentiation	GO:0002244	DNAI4	79819
hematopoietic progenitor cell differentiation	GO:0002244	DOCK1	1793
hematopoietic progenitor cell differentiation	GO:0002244	DOCK7	85440
hematopoietic progenitor cell differentiation	GO:0002244	EEF2	1938
hematopoietic progenitor cell differentiation	GO:0002244	EML1	2009
hematopoietic progenitor cell differentiation	GO:0002244	EPHX2	2053
hematopoietic progenitor cell differentiation	GO:0002244	ESCO2	157570
hematopoietic progenitor cell differentiation	GO:0002244	EXT1	2131
hematopoietic progenitor cell differentiation	GO:0002244	FSD1	79187
hematopoietic progenitor cell differentiation	GO:0002244	FST	10468
hematopoietic progenitor cell differentiation	GO:0002244	FSTL3	10272
hematopoietic progenitor cell differentiation	GO:0002244	G6PC3	92579
hematopoietic progenitor cell differentiation	GO:0002244	GPATCH4	54865
hematopoietic progenitor cell differentiation	GO:0002244	HDAC1	3065
hematopoietic progenitor cell differentiation	GO:0002244	HDAC6	10013
hematopoietic progenitor cell differentiation	GO:0002244	HDAC7A	798603
hematopoietic progenitor cell differentiation	GO:0002244	HDAC9A	101885469
hematopoietic progenitor cell differentiation	GO:0002244	HEATR9	256957
hematopoietic progenitor cell differentiation	GO:0002244	HERC6	55008
hematopoietic progenitor cell differentiation	GO:0002244	HLX1	327096
hematopoietic progenitor cell differentiation	GO:0002244	HOXB3	3213
hematopoietic progenitor cell differentiation	GO:0002244	HRNR	388697
hematopoietic progenitor cell differentiation	GO:0002244	HYAL2	8692
hematopoietic progenitor cell differentiation	GO:0002244	IFT88	8100
hematopoietic progenitor cell differentiation	GO:0002244	ING4	51147
hematopoietic progenitor cell differentiation	GO:0002244	INHBA	3624
hematopoietic progenitor cell differentiation	GO:0002244	JMJD1CA	571586
hematopoietic progenitor cell differentiation	GO:0002244	KCNAB2	8514
hematopoietic progenitor cell differentiation	GO:0002244	KCP	375616
hematopoietic progenitor cell differentiation	GO:0002244	KDM2BB	562643

hematopoietic progenitor cell differentiation	GO:0002244	KIF3A	11127
hematopoietic progenitor cell differentiation	GO:0002244	KRT75	9119
hematopoietic progenitor cell differentiation	GO:0002244	KRTAP5-5	439915
hematopoietic progenitor cell differentiation	GO:0002244	MBD3B	321217
hematopoietic progenitor cell differentiation	GO:0002244	MBD6	114785
hematopoietic progenitor cell differentiation	GO:0002244	MGST1.1	449784
hematopoietic progenitor cell differentiation	GO:0002244	MIB1	57534
hematopoietic progenitor cell differentiation	GO:0002244	MIXL1	83881
hematopoietic progenitor cell differentiation	GO:0002244	MMP21	118856
hematopoietic progenitor cell differentiation	GO:0002244	MRGPRX1	259249
hematopoietic progenitor cell differentiation	GO:0002244	MUC19	283463
hematopoietic progenitor cell differentiation	GO:0002244	MUC4	4585
hematopoietic progenitor cell differentiation	GO:0002244	MYCB	393141
hematopoietic progenitor cell differentiation	GO:0002244	NAP1L4A	337155
hematopoietic progenitor cell differentiation	GO:0002244	NOTCH1A	30718
hematopoietic progenitor cell differentiation	GO:0002244	NOTCH1B	794892
hematopoietic progenitor cell differentiation	GO:0002244	NOTCH3	4854
hematopoietic progenitor cell differentiation	GO:0002244	NPAS4L	108449885
hematopoietic progenitor cell differentiation	GO:0002244	PDGFRA	5156
hematopoietic progenitor cell differentiation	GO:0002244	PKD2	5311
hematopoietic progenitor cell differentiation	GO:0002244	PLD4	122618
hematopoietic progenitor cell differentiation	GO:0002244	PLEK	5341
hematopoietic progenitor cell differentiation	GO:0002244	PPP4R2	151987
hematopoietic progenitor cell differentiation	GO:0002244	PRDM12B	492816
hematopoietic progenitor cell differentiation	GO:0002244	PRDM16	63976
hematopoietic progenitor cell differentiation	GO:0002244	PRRC2C	23215
hematopoietic progenitor cell differentiation	GO:0002244	PSEN1	5663
hematopoietic progenitor cell differentiation	GO:0002244	PSEN2	5664
hematopoietic progenitor cell differentiation	GO:0002244	PTGER4A	562469
hematopoietic progenitor cell differentiation	GO:0002244	PTPN6	5777
hematopoietic progenitor cell differentiation	GO:0002244	PTPRC	5788
hematopoietic progenitor cell differentiation	GO:0002244	PTPRQ	374462
hematopoietic progenitor cell differentiation	GO:0002244	PTPRZ1	5803
hematopoietic progenitor cell differentiation	GO:0002244	PYGO1	26108
hematopoietic progenitor cell differentiation	GO:0002244	RBB4L	322129
hematopoietic progenitor cell differentiation	GO:0002244	RBM47	54502
hematopoietic progenitor cell differentiation	GO:0002244	RBMY	19657
hematopoietic progenitor cell differentiation	GO:0002244	REST	5978
hematopoietic progenitor cell differentiation	GO:0002244	RGD1307947	314788
hematopoietic progenitor cell differentiation	GO:0002244	RRS1	23212
hematopoietic progenitor cell differentiation	GO:0002244	SAMD9	54809
hematopoietic progenitor cell differentiation	GO:0002244	SAMD9L	219285
hematopoietic progenitor cell differentiation	GO:0002244	SBDS	51119
hematopoietic progenitor cell differentiation	GO:0002244	SERPINB12	89777
hematopoietic progenitor cell differentiation	GO:0002244	SERPINB9F	20709
hematopoietic progenitor cell differentiation	GO:0002244	SETD1BA	567970
hematopoietic progenitor cell differentiation	GO:0002244	SFRP1	6422
hematopoietic progenitor cell differentiation	GO:0002244	SIGLEC10	89790
hematopoietic progenitor cell differentiation	GO:0002244	SIGLECG	243958
hematopoietic progenitor cell differentiation	GO:0002244	SIN3A	25942
hematopoietic progenitor cell differentiation	GO:0002244	SIPA1L3	23094
hematopoietic progenitor cell differentiation	GO:0002244	SIRPA	140885
hematopoietic progenitor cell differentiation	GO:0002244	SIRT7	51547
hematopoietic progenitor cell differentiation	GO:0002244	SLC7A6OS	84138
hematopoietic progenitor cell differentiation	GO:0002244	SLC8A3	6547
hematopoietic progenitor cell differentiation	GO:0002244	SMARCD1	6602
hematopoietic progenitor cell differentiation	GO:0002244	SMARCD2	6603
hematopoietic progenitor cell differentiation	GO:0002244	SMPD3	55512
hematopoietic progenitor cell differentiation	GO:0002244	SRSF4	6429
hematopoietic progenitor cell differentiation	GO:0002244	SSBP3	23648
hematopoietic progenitor cell differentiation	GO:0002244	STON2	85439
hematopoietic progenitor cell differentiation	GO:0002244	SUV39H1B	326906
hematopoietic progenitor cell differentiation	GO:0002244	TCAF2	285966
hematopoietic progenitor cell differentiation	GO:0002244	TENT2	167153
hematopoietic progenitor cell differentiation	GO:0002244	TGFB1	7040
hematopoietic progenitor cell differentiation	GO:0002244	TGFB1B	563884
hematopoietic progenitor cell differentiation	GO:0002244	THSD1	55901

hematopoietic progenitor cell differentiation	GO:0002244	TMEM143	55260
hematopoietic progenitor cell differentiation	GO:0002244	TMEM190	147744
hematopoietic progenitor cell differentiation	GO:0002244	TMEM91	641649
hematopoietic progenitor cell differentiation	GO:0002244	TNFRSF13B	23495
hematopoietic progenitor cell differentiation	GO:0002244	TOP2A	7153
hematopoietic progenitor cell differentiation	GO:0002244	TP53	7157
hematopoietic progenitor cell differentiation	GO:0002244	TRP53	22059
hematopoietic progenitor cell differentiation	GO:0002244	VMN1R13	113862
hematopoietic progenitor cell differentiation	GO:0002244	VMN1R214	171248
hematopoietic progenitor cell differentiation	GO:0002244	VMN2R74	546980
hematopoietic progenitor cell differentiation	GO:0002244	VOM1R71	494243
hematopoietic progenitor cell differentiation	GO:0002244	VOM1R86	494247
hematopoietic progenitor cell differentiation	GO:0002244	WDR38	401551
hematopoietic progenitor cell differentiation	GO:0002244	WDR7	23335
hematopoietic progenitor cell differentiation	GO:0002244	WNT16	51384
hematopoietic progenitor cell differentiation	GO:0002244	ZBTB24	9841
hematopoietic progenitor cell differentiation	GO:0002244	ZFAT	57623
hematopoietic progenitor cell differentiation	GO:0002244	ZFP784	654801
hematopoietic progenitor cell differentiation	GO:0002244	ZFP865	319748
hematopoietic progenitor cell differentiation	GO:0002244	ZFP980	100041379
hematopoietic progenitor cell differentiation	GO:0002244	ZNF784	147808
hematopoietic progenitor cell differentiation	GO:0002244	ZSWIM9	374920
hematopoietic stem cell differentiation	GO:0060218	ACE	1636
hematopoietic stem cell differentiation	GO:0060218	ADTRP1	550414
hematopoietic stem cell differentiation	GO:0060218	AK2	204
hematopoietic stem cell differentiation	GO:0060218	AP2A1	160
hematopoietic stem cell differentiation	GO:0060218	BATF	10538
hematopoietic stem cell differentiation	GO:0060218	BIF1.1	103908654
hematopoietic stem cell differentiation	GO:0060218	CDK6	1021
hematopoietic stem cell differentiation	GO:0060218	CHD2	1106
hematopoietic stem cell differentiation	GO:0060218	CNR2	1269
hematopoietic stem cell differentiation	GO:0060218	CSF3A	100270759
hematopoietic stem cell differentiation	GO:0060218	CSF3B	100190920
hematopoietic stem cell differentiation	GO:0060218	CXCL8A	100002946
hematopoietic stem cell differentiation	GO:0060218	DLC	30120
hematopoietic stem cell differentiation	GO:0060218	DLD	1738
hematopoietic stem cell differentiation	GO:0060218	EPAS1A	566886
hematopoietic stem cell differentiation	GO:0060218	EPAS1B	555192
hematopoietic stem cell differentiation	GO:0060218	ERCC2	2068
hematopoietic stem cell differentiation	GO:0060218	EXT1	2131
hematopoietic stem cell differentiation	GO:0060218	FEV	54738
hematopoietic stem cell differentiation	GO:0060218	FOSAB	394198
hematopoietic stem cell differentiation	GO:0060218	GATA2B	436962
hematopoietic stem cell differentiation	GO:0060218	HAL	3034
hematopoietic stem cell differentiation	GO:0060218	HDAC1	3065
hematopoietic stem cell differentiation	GO:0060218	HIF1AA	797150
hematopoietic stem cell differentiation	GO:0060218	HIF1AB	393202
hematopoietic stem cell differentiation	GO:0060218	HOXB4	3214
hematopoietic stem cell differentiation	GO:0060218	IL6	3569
hematopoietic stem cell differentiation	GO:0060218	JAG1A	140421
hematopoietic stem cell differentiation	GO:0060218	LMBR1L	55716
hematopoietic stem cell differentiation	GO:0060218	LRSAM1	90678
hematopoietic stem cell differentiation	GO:0060218	MEOX1	4222
hematopoietic stem cell differentiation	GO:0060218	METAP2B	323452
hematopoietic stem cell differentiation	GO:0060218	METTL22	79091
hematopoietic stem cell differentiation	GO:0060218	MIB1	57534
hematopoietic stem cell differentiation	GO:0060218	MIR125B1	406911
hematopoietic stem cell differentiation	GO:0060218	MIR125B-1	387236
hematopoietic stem cell differentiation	GO:0060218	MIR125B2	406912
hematopoietic stem cell differentiation	GO:0060218	MIR125B-2	723952
hematopoietic stem cell differentiation	GO:0060218	MIR126A	387145
hematopoietic stem cell differentiation	GO:0060218	MIR130A	406919
hematopoietic stem cell differentiation	GO:0060218	MIR142A	100033664
hematopoietic stem cell differentiation	GO:0060218	MIR155	406947
hematopoietic stem cell differentiation	GO:0060218	MIR181C	406957
hematopoietic stem cell differentiation	GO:0060218	MIR193B	574455
hematopoietic stem cell differentiation	GO:0060218	MIR542	664617

hematopoietic stem cell differentiation	GO:0060218	MIR99A	407055
hematopoietic stem cell differentiation	GO:0060218	MIRLET7E	406887
hematopoietic stem cell differentiation	GO:0060218	MLLT3	4300
hematopoietic stem cell differentiation	GO:0060218	MYCA	30686
hematopoietic stem cell differentiation	GO:0060218	NCOR2	9612
hematopoietic stem cell differentiation	GO:0060218	NOP14	8602
hematopoietic stem cell differentiation	GO:0060218	NOS1	4842
hematopoietic stem cell differentiation	GO:0060218	NOTCH1A	30718
hematopoietic stem cell differentiation	GO:0060218	NOTCH1B	794892
hematopoietic stem cell differentiation	GO:0060218	NPAS4L	108449885
hematopoietic stem cell differentiation	GO:0060218	PDCD2	5134
hematopoietic stem cell differentiation	GO:0060218	PDGFRB	5159
hematopoietic stem cell differentiation	GO:0060218	PLCG1	5335
hematopoietic stem cell differentiation	GO:0060218	PRDM16	63976
hematopoietic stem cell differentiation	GO:0060218	RPS29	6235
hematopoietic stem cell differentiation	GO:0060218	RSPO1	284654
hematopoietic stem cell differentiation	GO:0060218	SF3A3	10946
hematopoietic stem cell differentiation	GO:0060218	SF3B1	23451
hematopoietic stem cell differentiation	GO:0060218	SFRP1	6422
hematopoietic stem cell differentiation	GO:0060218	SNRNP70	6625
hematopoietic stem cell differentiation	GO:0060218	SP7	121340
hematopoietic stem cell differentiation	GO:0060218	SRF	6722
hematopoietic stem cell differentiation	GO:0060218	TAL1	6886
hematopoietic stem cell differentiation	GO:0060218	TBX16	30264
hematopoietic stem cell differentiation	GO:0060218	TERC	7012
hematopoietic stem cell differentiation	GO:0060218	TGFB1A	359834
hematopoietic stem cell differentiation	GO:0060218	TGFB1B	563884
hematopoietic stem cell differentiation	GO:0060218	TGFB3	7043
hematopoietic stem cell differentiation	GO:0060218	TGFBR2B	30739
hematopoietic stem cell differentiation	GO:0060218	TNNT2A	58071
hematopoietic stem cell differentiation	GO:0060218	TP53	7157
hematopoietic stem cell differentiation	GO:0060218	TRP53	22059
hematopoietic stem cell differentiation	GO:0060218	UFL1	23376
hematopoietic stem cell differentiation	GO:0060218	VHL	7428
hematopoietic stem cell differentiation	GO:0060218	WDR43	23160
hematopoietic stem cell differentiation	GO:0060218	WNT16	51384
hematopoietic stem cell differentiation	GO:0060218	WNT9A	7483
hematopoietic stem cell differentiation	GO:0060218	XRCC5	7520
lymphoid progenitor cell differentiation	GO:0002320	BATF	10538
lymphoid progenitor cell differentiation	GO:0002320	BCL2	596
lymphoid progenitor cell differentiation	GO:0002320	BMP4	652
lymphoid progenitor cell differentiation	GO:0002320	FLT3	2322
lymphoid progenitor cell differentiation	GO:0002320	IRF4A	100002070
lymphoid progenitor cell differentiation	GO:0002320	KIT	3815
lymphoid progenitor cell differentiation	GO:0002320	LY6E	4061
lymphoid progenitor cell differentiation	GO:0002320	SHH	6469
lymphoid progenitor cell differentiation	GO:0002320	SPI1	6688
lymphoid progenitor cell differentiation	GO:0002320	TREX1	11277
myeloid progenitor cell differentiation	GO:0002318	BRAF	673
myeloid progenitor cell differentiation	GO:0002318	FLT3	2322
myeloid progenitor cell differentiation	GO:0002318	JAM3	83700
myeloid progenitor cell differentiation	GO:0002318	KIT	3815
myeloid progenitor cell differentiation	GO:0002318	MLF1	4291
myeloid progenitor cell differentiation	GO:0002318	RUNX1	861
myeloid progenitor cell differentiation	GO:0002318	SLC37A4	2542
myeloid progenitor cell differentiation	GO:0002318	TET2	54790
negative regulation of hematopoietic progenitor cell differentiation	GO:1901533	METTL14	57721
negative regulation of hematopoietic progenitor cell differentiation	GO:1901533	MIXL1	83881
negative regulation of hematopoietic progenitor cell differentiation	GO:1901533	NFKBIAA	406463
negative regulation of hematopoietic stem cell differentiation	GO:1902037	ARF79F	40506
negative regulation of hematopoietic stem cell differentiation	GO:1902037	ASRIJ	37637
negative regulation of hematopoietic stem cell differentiation	GO:1902037	C1GALTA	34215
negative regulation of hematopoietic stem cell differentiation	GO:1902037	F2R	2149
negative regulation of hematopoietic stem cell differentiation	GO:1902037	HSPA9	3313
negative regulation of hematopoietic stem cell differentiation	GO:1902037	IRF7	3665
negative regulation of hematopoietic stem cell differentiation	GO:1902037	N4BP2L2	10443
negative regulation of hematopoietic stem cell differentiation	GO:1902037	NFE2L2	4780

negative regulation of hematopoietic stem cell differentiation	GO:1902037	OCIAD1	54940
negative regulation of hematopoietic stem cell differentiation	GO:1902037	OCIAD2	132299
negative regulation of lymphoid progenitor cell differentiation	GO:1905457	BLOC1S2	282991
negative regulation of myeloid progenitor cell differentiation	GO:1905454	DPF2	5977
negative regulation of pro-B cell differentiation	GO:2000974	HES1	3280
negative regulation of pro-B cell differentiation	GO:2000974	HES5	388585
negative regulation of pro-B cell differentiation	GO:2000974	NOTCH1	4851
positive regulation of hematopoietic progenitor cell differentiation	GO:1901534	CSF3R	1441
positive regulation of hematopoietic progenitor cell differentiation	GO:1901534	DHX36	170506
positive regulation of hematopoietic progenitor cell differentiation	GO:1901534	FLT1	2321
positive regulation of hematopoietic progenitor cell differentiation	GO:1901534	FOXC1	2296
positive regulation of hematopoietic progenitor cell differentiation	GO:1901534	KITLG	4254
positive regulation of hematopoietic progenitor cell differentiation	GO:1901534	MYD88	4615
positive regulation of hematopoietic progenitor cell differentiation	GO:1901534	NELFB	25920
positive regulation of hematopoietic progenitor cell differentiation	GO:1901534	NELFE	7936
positive regulation of hematopoietic progenitor cell differentiation	GO:1901534	SUPT5H	6829
positive regulation of hematopoietic progenitor cell differentiation	GO:1901534	TLR4BB	403132
positive regulation of hematopoietic progenitor cell differentiation	GO:1901534	TNFRSF1B	7133
positive regulation of hematopoietic stem cell differentiation	GO:1902038	BLOC1S2	282991
positive regulation of hematopoietic stem cell differentiation	GO:1902038	DDX46	9879
positive regulation of hematopoietic stem cell differentiation	GO:1902038	FOXC1	2296
positive regulation of hematopoietic stem cell differentiation	GO:1902038	NELFB	25920
positive regulation of hematopoietic stem cell differentiation	GO:1902038	NELFE	7936
positive regulation of hematopoietic stem cell differentiation	GO:1902038	SUPT5H	6829
positive regulation of myeloid progenitor cell differentiation	GO:1905455	BLOC1S2	282991
positive regulation of pro-B cell differentiation	GO:2000975	NUDT21	11051
positive regulation of pro-T cell differentiation	GO:2000176	ZBTB1	22890
pro-B cell differentiation	GO:0002328	FLT3	2322
pro-B cell differentiation	GO:0002328	LIG4	3981
pro-B cell differentiation	GO:0002328	PRKDC	5591
pro-B cell differentiation	GO:0002328	SOX4	6659
pro-B cell differentiation	GO:0002328	XRCC4	7518
pro-T cell differentiation	GO:0002572	FLT3	2322
pro-T cell differentiation	GO:0002572	GATA3	2625
regulation of hematopoietic progenitor cell differentiation	GO:1901532	ADORA2B	136
regulation of hematopoietic progenitor cell differentiation	GO:1901532	CSF3R	1441
regulation of hematopoietic progenitor cell differentiation	GO:1901532	EIF2AK2	5610
regulation of hematopoietic progenitor cell differentiation	GO:1901532	KDR	3791
regulation of hematopoietic progenitor cell differentiation	GO:1901532	MYD88	4615
regulation of hematopoietic progenitor cell differentiation	GO:1901532	NFKBIAA	406463
regulation of hematopoietic progenitor cell differentiation	GO:1901532	PDCD2	5134
regulation of hematopoietic progenitor cell differentiation	GO:1901532	PDGFRA	5156
regulation of hematopoietic progenitor cell differentiation	GO:1901532	TLR4BB	403132
regulation of hematopoietic progenitor cell differentiation	GO:1901532	TNFRSF1B	7133
regulation of hematopoietic progenitor cell differentiation	GO:1901532	VEGFA	7422
regulation of hematopoietic stem cell differentiation	GO:1902036	ABL1	25
regulation of hematopoietic stem cell differentiation	GO:1902036	ADORA2B	136
regulation of hematopoietic stem cell differentiation	GO:1902036	AP2A2	161
regulation of hematopoietic stem cell differentiation	GO:1902036	CBFB	865
regulation of hematopoietic stem cell differentiation	GO:1902036	CDCA7A	550236
regulation of hematopoietic stem cell differentiation	GO:1902036	CDK6	1021
regulation of hematopoietic stem cell differentiation	GO:1902036	CRHB	492507
regulation of hematopoietic stem cell differentiation	GO:1902036	DLD	1738
regulation of hematopoietic stem cell differentiation	GO:1902036	DNMT3BB.1	317744
regulation of hematopoietic stem cell differentiation	GO:1902036	EIF2AK2	5610
regulation of hematopoietic stem cell differentiation	GO:1902036	F11R.1	323696
regulation of hematopoietic stem cell differentiation	GO:1902036	FGF10A	359830
regulation of hematopoietic stem cell differentiation	GO:1902036	FGFR2	2263
regulation of hematopoietic stem cell differentiation	GO:1902036	FGFR3	2261
regulation of hematopoietic stem cell differentiation	GO:1902036	FGFR4	2264
regulation of hematopoietic stem cell differentiation	GO:1902036	GATA1	2623
regulation of hematopoietic stem cell differentiation	GO:1902036	GATA2	2624
regulation of hematopoietic stem cell differentiation	GO:1902036	GATA3	2625
regulation of hematopoietic stem cell differentiation	GO:1902036	ITCH	83737
regulation of hematopoietic stem cell differentiation	GO:1902036	KMT2A	4297
regulation of hematopoietic stem cell differentiation	GO:1902036	LDB1	8861
regulation of hematopoietic stem cell differentiation	GO:1902036	LMO1	4004

regulation of hematopoietic stem cell differentiation	GO:1902036	LMO2	4005
regulation of hematopoietic stem cell differentiation	GO:1902036	METTL3	56339
regulation of hematopoietic stem cell differentiation	GO:1902036	MYB	4602
regulation of hematopoietic stem cell differentiation	GO:1902036	NR3C1	2908
regulation of hematopoietic stem cell differentiation	GO:1902036	OSM	5008
regulation of hematopoietic stem cell differentiation	GO:1902036	POMCA	353221
regulation of hematopoietic stem cell differentiation	GO:1902036	POMCB	100034412
regulation of hematopoietic stem cell differentiation	GO:1902036	PRKDC	5591
regulation of hematopoietic stem cell differentiation	GO:1902036	PSMA1	5682
regulation of hematopoietic stem cell differentiation	GO:1902036	PSMA2	5683
regulation of hematopoietic stem cell differentiation	GO:1902036	PSMA3	5684
regulation of hematopoietic stem cell differentiation	GO:1902036	PSMA4	5685
regulation of hematopoietic stem cell differentiation	GO:1902036	PSMA5	5686
regulation of hematopoietic stem cell differentiation	GO:1902036	PSMA6	5687
regulation of hematopoietic stem cell differentiation	GO:1902036	PSMA7	5688
regulation of hematopoietic stem cell differentiation	GO:1902036	PSMA8	143471
regulation of hematopoietic stem cell differentiation	GO:1902036	PSMB1	5689
regulation of hematopoietic stem cell differentiation	GO:1902036	PSMB10	5699
regulation of hematopoietic stem cell differentiation	GO:1902036	PSMB11	122706
regulation of hematopoietic stem cell differentiation	GO:1902036	PSMB2	5690
regulation of hematopoietic stem cell differentiation	GO:1902036	PSMB3	5691
regulation of hematopoietic stem cell differentiation	GO:1902036	PSMB4	5692
regulation of hematopoietic stem cell differentiation	GO:1902036	PSMB5	5693
regulation of hematopoietic stem cell differentiation	GO:1902036	PSMB6	5694
regulation of hematopoietic stem cell differentiation	GO:1902036	PSMB7	5695
regulation of hematopoietic stem cell differentiation	GO:1902036	PSMB8	5696
regulation of hematopoietic stem cell differentiation	GO:1902036	PSMB9	5698
regulation of hematopoietic stem cell differentiation	GO:1902036	PSMC1	5700
regulation of hematopoietic stem cell differentiation	GO:1902036	PSMC2	5701
regulation of hematopoietic stem cell differentiation	GO:1902036	PSMC3	5702
regulation of hematopoietic stem cell differentiation	GO:1902036	PSMC4	5704
regulation of hematopoietic stem cell differentiation	GO:1902036	PSMC5	5705
regulation of hematopoietic stem cell differentiation	GO:1902036	PSMC6	5706
regulation of hematopoietic stem cell differentiation	GO:1902036	PSMD1	5707
regulation of hematopoietic stem cell differentiation	GO:1902036	PSMD10	5716
regulation of hematopoietic stem cell differentiation	GO:1902036	PSMD11	5717
regulation of hematopoietic stem cell differentiation	GO:1902036	PSMD12	5718
regulation of hematopoietic stem cell differentiation	GO:1902036	PSMD13	5719
regulation of hematopoietic stem cell differentiation	GO:1902036	PSMD14	10213
regulation of hematopoietic stem cell differentiation	GO:1902036	PSMD2	5708
regulation of hematopoietic stem cell differentiation	GO:1902036	PSMD3	5709
regulation of hematopoietic stem cell differentiation	GO:1902036	PSMD4	5710
regulation of hematopoietic stem cell differentiation	GO:1902036	PSMD5	5711
regulation of hematopoietic stem cell differentiation	GO:1902036	PSMD6	9861
regulation of hematopoietic stem cell differentiation	GO:1902036	PSMD7	5713
regulation of hematopoietic stem cell differentiation	GO:1902036	PSMD8	5714
regulation of hematopoietic stem cell differentiation	GO:1902036	PSMD9	5715
regulation of hematopoietic stem cell differentiation	GO:1902036	PSME1	5720
regulation of hematopoietic stem cell differentiation	GO:1902036	PSME2	5721
regulation of hematopoietic stem cell differentiation	GO:1902036	PSME3	10197
regulation of hematopoietic stem cell differentiation	GO:1902036	PSME4	23198
regulation of hematopoietic stem cell differentiation	GO:1902036	PSMF1	9491
regulation of hematopoietic stem cell differentiation	GO:1902036	PUS7	54517
regulation of hematopoietic stem cell differentiation	GO:1902036	RUNX1	861
regulation of hematopoietic stem cell differentiation	GO:1902036	SETD1A	9739
regulation of hematopoietic stem cell differentiation	GO:1902036	TAL1	6886
regulation of hematopoietic stem cell differentiation	GO:1902036	TCF12	6938
regulation of hematopoietic stem cell differentiation	GO:1902036	TCF3	6929
regulation of hematopoietic stem cell differentiation	GO:1902036	TP73	7161
regulation of hematopoietic stem cell differentiation	GO:1902036	TPH1A	352943
regulation of hematopoietic stem cell differentiation	GO:1902036	TPH1B	415103
regulation of hematopoietic stem cell differentiation	GO:1902036	TPH2	121278
regulation of hematopoietic stem cell differentiation	GO:1902036	URB2	9816
regulation of hematopoietic stem cell differentiation	GO:1902036	WNT16	51384
regulation of hematopoietic stem cell differentiation	GO:1902036	YAP1	10413
regulation of hematopoietic stem cell differentiation	GO:1902036	YTHDF2	51441
regulation of lymphoid progenitor cell differentiation	GO:1905456	ANKLE1	126549

regulation of myeloid progenitor cell differentiation	GO:1905453	ANKLE1	126549
regulation of pro-B cell differentiation	GO:2000973	FLCN	201163
regulation of pro-B cell differentiation	GO:2000973	FNIP1	96459
regulation of pro-B cell differentiation	GO:2000973	SOS1	6654
regulation of pro-B cell differentiation	GO:2000973	SOS2	6655
