# Supplementary material for: Ginsenoside Rg1 as a Potential Regulator of Hematopoietic Stem/Progenitor Cells
Source: Stem Cells Int. 2021 Dec 31;2021:4633270. doi: 10.1155/2021/4633270 (PMC8741398; doi:10.1155/2021/4633270)
Supplement: Supplementary Materials — Supplementary Material 1: putative targets of Rg1 identified using TargetNet. Supplementary Material 2: putative targets of Rg1 identified using SwissTargetPrediction. Supplementary Material 3: genes involved in HSC proliferation. Supplementary Material 4: genes involved in HSC migration. Supplementary Material 5: genes involved in HSC differentiation. Supplementary Material 6: genes involved in HPC differentiation. Supplementary Material 7: databases used in this study. [file 4633270.f1.zip › Supplementary Material 7.pdf]

**The links of databases used in our study.**

| Plantform Name | Links |
|---|---|
| TargetNet | http://targetnet.scbdd.com/ |
| SwissTarget | http://www.swisstargetprediction.ch/ |
| Comparative Toxicogenomics Database | http://ctdbase.org/ |
| Metascape | https://metascape.org/gp/index.html#/main/step1 |
